# Relationship between alcohol drinking and arterial hypertension in indigenous people of the Mura ethnics, Brazil

**DOI:** 10.1371/journal.pone.0182352

**Published:** 2017-08-04

**Authors:** Alaidistania Aparecida Ferreira, Zilmar Augusto Souza-Filho, Maria Jacirema F. Gonçalves, Juliano Santos, Angela Maria G. Pierin

**Affiliations:** 1 Escola de Enfermagem de Manaus, Universidade Federal do Amazonas, Manaus, Amazonas, Brazil; 2 Instituto Leônidas e Maria Deane, Fundação Oswaldo Cruz, Manaus, Amazonas, Brazil; 3 Escola de Enfermagem da Universidade de São Paulo, São Paulo, Brazil; Medizinische Fakultat der RWTH Aachen, GERMANY

## Abstract

**Objective:**

To identify the consumption of alcoholic beverage and the relation with hypertension, their prevalence and associated factors, in indigenous Mura, Brazil.

**Methods:**

A cross-sectional population-based study was conducted with 455 adult indigenous aged 18 years or more of Mura ethnics in Amazonia, Brazil. Interview was conducted and the alcohol intake was assessed by the Alcohol Use Disorders Identification Test. Blood pressure was measured in three measurements and the mean of the last two measurements was used. Physical examination included the following data: weight, height, waist and neck circumference, bioimpedance, and capillary measurement of glucose, triglycerides and cholesterol. Through multivariate Logistic regression in stepwise, the odds ratios for alcohol consumption and associated factors were identified.

**Results:**

The prevalence of alcoholic beverage was 40.2%, with no significant difference for hypertension in those who drink (23.0%) and those who did not drink (29.0%). Referred hypertension in indigenous was associated to less use of alcoholic beverages (14.2% vs 24.3%, P = 0.009). After an adjusted analysis (Odds Ratio, 95% CI), there was a positive association between alcoholic drink intake and male sex (10.27, CI: 5.76–18.30), smoking (4.72, CI: 2.35–9.46) and live in rural areas (9.77, CI: 5.08–18.79). On the other hand, age (0.95, IC: 0.94–0.97), and absence of dyslipidemia (0.41, CI: 0.19–0.89) were associated to lower alcohol consumption.

**Conclusion:**

The prevalence of alcoholic beverage was high and associated with referred hypertension, but this association was not maintained after adjusted analysis. Changes to habits and inappropriate lifestyles in indigenous populations and living in urban areas may contribute to increase risk for cardiovascular diseases. Therefore, health policies should be implemented to meet the uniqueness of indigenous people.

## Introduction

Epidemiological and demographic changes have occurred in the indigenous population of Brazil, which has high socio-cultural, economic and political diversity [1, 2]. From this, there were transformations in the practices of subsistence, food, and physical activity and in the indigenous culture itself. Adoption of inadequate habits and lifestyles by indigenous populations increases the risks for diseases, especially chronic non communicable diseases, such as hypertension, and social impact diseases such as alcoholism [3].

In the general population, hypertension and alcoholism are major public health problems in the chronic diseases. Both are multifactorial diseases, of high prevalence and considered a risk factor for other diseases [4].

The studies carried out in the 1970s and 1980s in Brazil showed that arterial hypertension was practically non-existent in indigenous populations [5–9]. However, this scenario has changed. A systematic review and meta-analysis of Brazilian indigenous populations showed that the pooled prevalence of hypertension in the period from 1970 to 2014 was 6.2% (95% CI: 3.1% - 10.3%). In the regression, the value of the odds ratio was 1.12 (95% CI: 1.07–1.18, P <0.001), indicating an increase of 12% per year in the odds of an indigenous person presenting arterial hypertension [10].

Alcohol is the drug most commonly used in the life of Brazil's general population. Data from the National Survey of Alcohol and Drugs of 2012 indicated that 50.0% of the people evaluated consumed alcohol in the last 12 months and 39% consumed five or more doses when they drank [11]. In the indigenous population the drinking of alcoholic beverage has also been outstanding. In indigenous Potiguaras of the state of Paraíba, northeastern Brazil, the prevalence of alcohol consumption was 41.8% [12]; In the Indigenous Community of Central Brazil the alcohol consumption was lower (19%), although that is one research that aims to find hypertension prevalence (29.5%), it is important emphasize that they live in areas with access to urban areas [13], such as indigenous of present study who live in urban areas or next to. This way, they are changing their culture and life habits, which can modify cardiovascular risk factors.

It is, therefore, considered that alcoholism and hypertension in indigenous populations of Brazil are worrying and increasing problems, as stated above [10]. In the same way, other qualitative studies highlight that indigenous are getting easy access to industrialized alcohol, what impact in their consumption [3, 12, 14]. It would be desirable that indigenous have no problems with alcohol and hypertension, because both problems are related to lifestyles. However, we did not identify a study with the Mura ethnic group, focusing on these two diseases and associated factors. Indigenous Mura inhabit the Amazon Region, which has the largest proportion of indigenous people of the country, especially those still without or with little contact with non-indigenous peoples. Thus, the present study aimed to identify the consumption of alcoholic beverages and the relationship with hypertension, their prevalence and associated factors, in indigenous Mura in Brazil.

## Methods

### Study design and context

Cross-sectional, population-based epidemiological study on alcoholism and hypertension among indigenous Mura in Amazonia, Brazil. This is one of the most populated indigenous ethnics in Brazil who live in the western region of the state of Amazonas. The highest concentration of these indigenous people is in the municipality of Autazes, where the study is carried out, whose geographical access is mixed, part of the route is terrestrial and part is fluvial. The Manaus Indigenous Special Sanitary District, that take care indigenous health, in the municipality of Autazes is organized in two base poles: Pantaleão and Murutinga. The base pole Pantaleão is located in the urban perimeter and the base pole Murutinga, in the rural zone, in the Murutinga’s village. One village was selected in each base pole for the field collection, the urban contingent was formed by indigenous residents in the urban area of Autazes, enrolled and followed by the health team who works in the Pantaleão base pole, and the indigenous participants of the rural zone, were the residents of the village Murutinga, where the Murutinga base pole is located.

The study was approved by the Research Ethics Committee (CAAE USP N° 34559614.0.0000.5392–09/23/2014), National Commission for Research Ethics (CAAE CONEP N° 45277015.3.0000.5392–09/18/2015) and received the authorization of the Admission In Indigenous Lands of the National Indigenous Foundation (FUNAI) (127 / AAEP / PRES / 2015 19/10/2015). After all the explanations about the procedures of the study, all the indigenous that accepted to participate signed the Free and Informed Consent Form.

### Participants

Hypertension prevalence rates considered as reference value for the sample size calculation was 29.7%, based on data from indigenous people in the village of Jaguapirú [13]. Assuming a prevalence of 30% with accuracy of 5%, confidence interval (CI) of 95% and a power of 80%, the required sample size for finite population was estimated at 425 individuals. The sample size was added of 10% for losses and refusals, totaling 477 individuals.

We recruited 455 participants (221 of the urban area and 234 of the rural area). The participants were selected by simple random sampling of the household, with a subsequent draw among the recruited participants, during domiciliar visit. The active search of the indigenous adults in the respective households was carried out with the collaboration of the Indigenous Health Agents of the Base Poles. The criteria for inclusion in the study were: self-declaration as indigenous, according to indigenous birth registration (RANI) issued by the National Indigenous Foundation (FUNAI); age greater than or equal to 18 years; and speak the Portuguese language. The exclusion criteria were: pregnant women, non-residents in the selected village and those with difficulties in verbal communication.

### Data sources

Data collection took place from January to March 2016, by two duly trained nurses researchers who conducted an individual interview and health evaluation of the participants, in which he variables described below were evaluated (S1 Appendix):

1. Demographic, socioeconomic and eating habits variables were evaluated with an instrument adapted from the National Survey of Health and Nutrition of Indigenous Peoples [15]. For income categorization we used the minimum wage adopted in Brazil in June 2016, which was equivalent to approximately US $ 230,00 per month. Persons who receive from one to two minimum wage per month (US $ 230,00 to US $ 460,00) or those who receive three or more minimum wage (US $ 690,00).
2. Variables on habits and lifestyles including food consumption, smoking, physical activity and alcoholic beverage were gathered. Dietary habits were evaluated based on the adaptation of the National Survey of Health and Nutrition of Indigenous Peoples [15], food classification [16] and frequency of food consumption [17], obtaining a maximum score of 15 points, as the following detail: almost never / never = 1 point; one to two days = 2 points; three to four days = 3 points; five to six days = 4 points; every day = 5 points. The referred food score was used for food consumption in natura, consumption of semi-processed foods, and consumption of industrialized products, according to food classification. Alcohol intake was assessed using the Brazilian version of the *Alcohol Use Disorders Identification Test* (AUDIT) [18], which aims at the early detection and identification of risk groups and the screen problematic alcohol use and its consequences in the last year to make interventions, in clinical samples and in the general population. It consists of 10 questions that explore the use, dependence and problems related to alcohol use. The score ranges from 0 to 40, but when it reaches eight or more, it indicates the need for a more specific diagnostic investigation. We classified participants according to AUDIT if they were at risk (yes) or at not at risk for alcohol abuse (no), and so used this variable as outcome. The first AUDIT's question is about the frequency of consumption and is answered on a scale ranging from 0 (Never) to 4 (Four or more times per week). The answer "never" classifies the participant as not consuming alcohol.
3. Variables on personal and family history, hypertension and other comorbidities were assessed.

### Blood pressure measurement

The blood pressure measurement was performed with digital arm automatic device validated [19] and properly calibrated. Three blood pressure measurements were performed, with a five-minute interval between the three measurements, under the following steps: previous rest of 10 minutes, placement of the participant sitting comfortably, feet straight and supported on the floor, relaxed back and arm properly supported and at the level of the heart. It was asked if the participant did not have a full bladder without using coffee, smoke and alcohol 30 minutes and physical activity at least 60 minutes before blood pressure measurement. A cuff suitable for arm size was used.

Hypertension used to calculate prevalence was defined as systolic blood pressure ≥140 and / or diastolic blood pressure ≥90 mmHg, or self-reported hypertension (referred), when indigenous reported having the diagnosis of hypertension done by a physician or a nurse or when a person take anti-hypertensive medicine, independently of the blood pressure values measured in the interview. Hypertension was classified in stages following the recommendations of the VII Brazilian Guidelines on Hypertension [20, 21].

### Anthropometric measurements

All the indigenous were submitted to anthropometric evaluation, body weight and height, wearing usual light clothing and barefoot. In height measurements, a stadiometer with a scale of 20 centimeter to 200 centimeter and a precision of 0.1 centimeter was used, fixed on a rigid wall, keeping the body erect looking at the horizon. The body mass index of the indigenous was evaluated according to World Health Organization [22]. The circumference of the neck and waist were measured with an inelastic tape measuring 2.00 meters, graduated from 0.5 in 0.5 centimeters. The circumference of the indigenous’s neck was directed to stand in an upright position, with the head positioned in the horizontal plane of Frankfort [23]. Regarding the classification of the circumference of the neck indicate an increased risk if the circumference of the neck is ≥37 cm for men and ≥34 cm for women [24, 25]. For the waist circumference the participant was asked to stand, with the abdomen relaxed and the arms laterally apart. The anthropometric tape was placed in the lower perimeter of the abdominal region, between the last costal arch and the upper border of the iliac crest following recommendation of the World Health Organization were used [22]. All these measures were used in continuous scale, comparing by means.

Bioimpedance was performed to measure the percentage of body fat, percentage of skeletal muscle, and visceral fat. Calibrated portable digital bioimpedance scale, with a maximum capacity of 150 kg was used [26]. The conicity index was determined using a mathematical formula with measures of weight, height and waist circumference, and the recommended cutoff point for high coronary risk is 1.25 for men and 1.18 for women [27], although we used this index in its continuous scale.

### Capillary glycemic measurement and lipid profile

The indigenous were instructed to remain fasted for eight hours and at a second visit were dosed glucose, triglycerides and total cholesterol, with the use of calibrated digital automatic portable device and use of reagent tapes. The blood samples were obtained with the use of a disposable puncture device (lancet), for individual use, in the digital pulp of the index finger. For classification were used the Guideline of the Brazilian Society of Diabetes Mellitus [28] and the Brazilian Guideline on Dyslipidemia and Prevention of Atherosclerosis [29].

For the evaluation of the indigenous health personal history, regarding to dyslipidemia, diabetes mellitus, stroke and heart problems, indigenous were asked if they were diagnosed with one of these conditions. Specifically on dyslipidemia, we asked if they were tested in the last year or if they use specific drugs to treat dyslipidemia, with the answers: yes, no or do not know.

#### Physical activity level

The International Physical Activity Questionnaire (IPAQ) has acceptable reliability and validity[30] and is used to estimate the overall physical activity level of an individual in metabolic equivalent (MET)-min/week by determining the duration (in minutes) and number of days (in 1 week) of engagement in three specific types of activity (walking, moderate-intensity activities, and high-intensity activities) across a comprehensive set of domains (leisure time, work-related and transport-related physical activities, and domestic and gardening activities) in the past 7 days. Respondents were classified according to two physical activity levels, namely sedentary (irregularly active and sedentary) and active (active and very active)

### Statistical analysis

The qualitative variables are presented by absolute frequency (n) and relative frequency (%), and the quantitative variables are presented by mean and standard deviation. In the bivariate analysis, the Pearson's Chi-square test or Fisher's test was used for the categorical variables. If the continuous variables were normal according to Shapiro Wilk's test, we used the two-tailed t-Student test to compare mean differences between groups, performing a priori tests for equality of variances. For non-parametric continuous variables we used Wilcoxon's test. The statistical significance level was P-value < = 0.05.

As a response variable, we used the AUDIT *Alcohol Use Disorders Identification Test score*, categorized at risk (yes) or at non-risk (no) for alcohol abuse, as described above. The multivariate Logistic model with robust variance was adjusted in stepwise forward method, with criterion to enter the modeling, the critical level of P≤0.20 values.

The modeling process started with empty model. Variable entered the multivariate regression at P <0.20 and were removed at P >0.10, but referred hypertension was kept in the model as an adjustment variable, because during the modeling this variable had significance changed by age. In the final model, variables with a significance level of less or equal 5% and those used as control variables were considered. The Odds Ratio (OR) was used as a measure of association, accompanied by a 95% confidence interval. The model Goodness-of-fit was evaluated by Hosmer & Lemeshow test. During the model adjustment procedure, the multicollinearity between the independent variables was evaluated using the inflation factor of the variance. Interactions between independent variables and potential confounders were also tested, such as sex, age and education.

## Results

A total of 455 indigenous Mura participated of the research. There was a non-response rate of 4.6% between the calculated sample and the subjects effectively recruited in the research. Most of the participants were adults with a mean age of 42.1 years (Standard Deviation: 16.7); the youngest and oldest participants were 18 and 81 years old, respectively, and only 7.7% were older than 70 years of age. The participants' educational level was low, and only 4.0% had higher education (data not shown). The monthly family income was less than US $ 230,00 for 37.6% of the families. The habit of cultivation is rare, because they have access to buy what they need. Only 28.6% of the participants declared formal and fixed job and 97.7% are beneficiary of government income transfer programme, called Bolsa Família (Family Grant).

**Fig 1:**
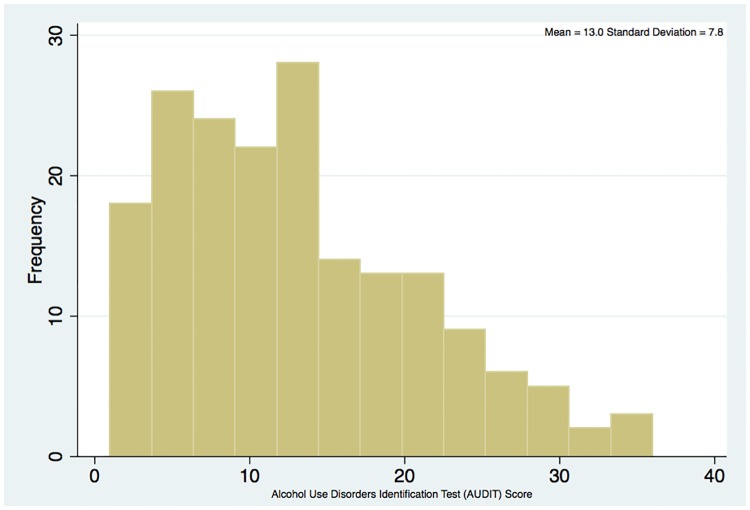
Distribution of *Alcohol Use Disorders Identification Test* (AUDIT) score among indigenous Mura—Brazil.

**Fig 2:**
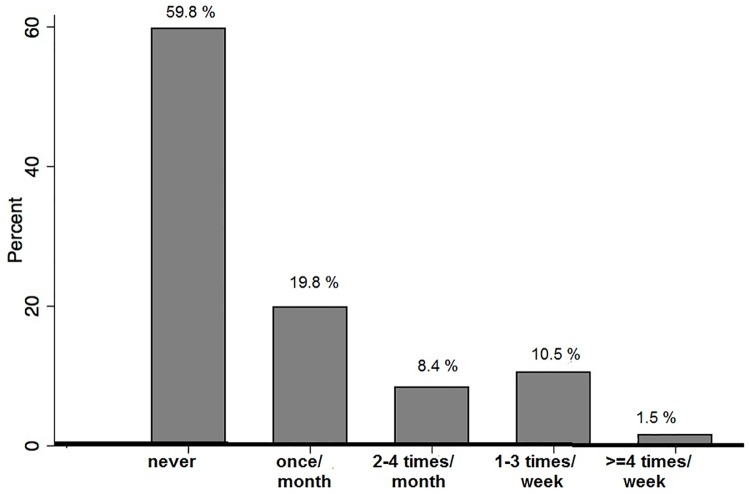
Frequency of alcohol consumption among indigenous Mura—Brazil.

The prevalence of alcohol use among the indigenous the indigenous population was 40.2%, according to AUDIT. The distribution of the AUDIT-scores is showed in Fig 1. Most indigenous people reported not drinking alcohol in the last 12 months (no risk), and those with risk most frequently have declared drinking alcohol at least once per month, after that, 10.5% declared drinking alcohol one to three times per week (Fig 2). For those with risk for problematic alcohol drinking, they reported have consumed 10 or more doses of alcohol (57.4%) (data not shown). As the indigenous have their own alcoholic beverage, we asked their habits e frequency of consumption, and we found 90.2% of indigenous declared never consumed their spirituous/craft beverages.

The biosocial, educational, and life habits variables of indigenous people associated (P <0.05) with alcohol intake were: sex, prevalence was higher in men; youngest age; be responsible for family income; have temporary paid work; living in the rural area; number of children; and sell agricultural products and fishing and to be retired and smoker are not significantly associated with alcohol abuse (Table 1).

**Table 1:** Biosocial, educational and life habits variables in relation to alcohol abuse of the indigenous Mura, Brazil.

Variables	Alcohol abuse				P-value^a^
	No		Yes		
	n	%	n	%	
**Place of residence**					
**Urban area**	172	77.8	49	22.2	**<0.001**
**Rural**	100	42.7	134	57.3	
**Sex**					
Female	205	75.5	58	31.5	**<0.001**
Male	67	24.5	125	68.5	
**Age (years)—Mean (SD)** **Minimun: 18; Maximun: 81**	44.2	(17.6)	38.9	(14.9)	**0.002**^b^
**Number of children—Mean (SD)**	4.6	(3.2)	3.85	(3.1)	**0.006**^b^
**Status Marital**					
Without partner	75	27.5	46	25.0	0.590
With partner	197	72.5	137	75.0	
**Education**					
Unlettered Fundamental incomplete (1st° to 5th° year)	191	70.3	122	66.5	0.470
Basic and Higher Education	81	29.7	61	33.5	
**Family income (minimum wage)**^c^					
1–2	232	94.0	162	93.0	0.840
≥ 3	15	6.0	12	7.0	
**Responsible for family income**					
**Participant**	143	52.6	133	60.7	**<0.001**
**Partner**	102	37.5	30	29.0	
**Others**	27	9.9	20	10.0	
**Temporary paid work**					
**Yes**	135	49.6	113	61.7	**0.013**
**No**	137	50.4	70	38.3	
**Sale of *agricultural* and *fishery* products**					
**Yes**	99	36.4	93	50.8	**0.003**
**No**	173	63.6	90	49.2	
**Sale of *crafts* and / or cultural production**					
**Yes**	12	4.5	03	1.6	**0.117**
**No**	260	95.5	180	8.4	
**Receives retirement**					
**Yes**	78	28.7	42	23.0	**0.194**
**No**	194	71.3	141	77.0	
**Smoker**					
**Yes**	35	12.9	58	31.7	**0.001**
**No**	171	62.9	81	44.3	
**Ex-smoker**	66	24.2	44	24.0	
**Physical activity**					
**Sedentary / Irregularly active**	126	46.3	89	47.3	0.634
**Active / Very active**	146	53.7	94	52.7	

SD = Standard Deviation;

^a^ Fisher's test

^b^ Wilcoxon's test

^c^ Minimum wage actual of R$ 880.00, equivalent to approximately US $ 230,00 in June, 2016.

The evaluation of the anthropometric data of the indigenous showed that alcohol abuse was significantly associated with: increased weight, height, neck circumference and skeletal muscle percentage; but the conicity index and percentage of body fat were lower. The total cholesterol was lower in those who consumed alcoholic beverages, but the consumption of industrialized products score was higher and consumption of *in natura* products score was lower. Having a personal history of diabetes mellitus, heart problem and dyslipidemia were more frequent in indigenous people who did not use alcohol (Table 2).

**Table 2:** Anthropometric variables, diet, lipid profile, blood glucose and health personal history according to alcohol abuse in indigenous Mura, Brazil.

	Alcohol abuse		
Variables	No Mean (SD)	Yes Mean (SD)	P value
**Weight** (kg)	62.95 (12.77)	66.46 (12.31)	**0.004**^b^
**Height** (cm)	152.78 (7.21)	159.02 (7.44)	**<0.001**^a^
**BMI** (kg/m^2^)	26.88 (5.01)	26.30 (4.38)	0.288^b^
**Conicity Index**	1.28 (0.08)	1.26 (0.07)	**0.007**^b^
**Neck Circumference (cm)**	35.95 (3.39)	37.78 (3.31)	**< 0.001**^b^
**Waist Circumference (cm)**	89.89 (11.32)	89.04 (11.27)	0.431^a^
**Body Fat**	35.66 (10.14)	28.15 (10.52)	**<0.001**^b^
**Skeletal muscle**	27.79 (6.01)	33.42 (6.96)	**<0.001**^b^
**Food consumption *in natura***	8.64 (6.34)	5.83 (5.30)	**<0.001**^b^
**Consumption of semi-processed foods**	6.37 (2.03)	6.32 (1.98)	0.694^b^
**Consumption of industrialized products**	5.85 (4.12)	7.14 (4.17)	**< 0.001**^b^
**Triglycerides (mg/dL)**	166.95 (111.98)	158.50 (93.13)	0.834^b^
**Total cholesterol (mg/dL)**	190.28 (33.74)	183.18 (29.59)	**0.009**^b^
**Blood Glucose (mg/dL)**	72.53 (25.84)	69.87 (19.67)	0.239^a^
**Personal background**	n (%)	n (%)	
Cardiac problem	21 (7.7)	9 (5.0)	**0.035**^c^
Diabetes mellitus	21 (7.7)	2 (1.1)	**<0.001**^c^
Stroke	9 (3.3)	6 (3.3)	>0.999^c^
Dislypidemias	49 (18.1)	18 (9.8)	**<0.001**^c^

^a^ t-student's test

^b^ Wilcoxon's test

^c^ Fisher's test

SD: Standard Deviation; BMI: Body Mass Index.

Table 3 shows the hypertension data of the indigenous Mura and their relationship with alcoholic beverage consumption. The prevalence of arterial hypertension was not different (P = 0.161) among those at risk of alcohol abuse (23%) and those with no risk (29%). We identified a statistically significant association between having a previous diagnosis of hypertension and no risk for problematic alcoholic consumption.

**Table 3:** Hypertension and alcohol abuse in the indigenous Mura, Brazil.

	Alcohol abuse				
	No	(n = 272)	Yes	(n = 183)	P-value
**Systolic pressure**—Mean (SD)	122.54	(22.38)	119.94	(17.26)	0.556^a^
**Diastolic pressure**—Mean (SD)	76.36	(9.61)	75.98	(10.10)	0.438^a^
**Previous diagnosis of hypertension (referred)**		%		%	
Yes	66	24.3	26	14.2	**0.009**^b^
No	206	75.7	157	85.8	
**Hypertension**^c^					
Yes	79	29.0	42	23.0	0.161^b^
No	193	71.0	141	77.0	
**Blood Pressure Classification**					
Normal	149	54.8	101	55.2	0.469^b^
Pre-hypertensive	76	27.9	56	30.6	
Hypertension stage 1	25	9.2	17	9.3	
Hypertension stage 2	13	8.8	7	3.8	
Hypertension stage 3	9	3.3	2	1.1	
**Last time measured blood pressure**					
< 1 month	73	51.8	27	38.6	0.074^b^
1 to 6 months	57	40.4	37	52.9	
6 to 12 months	7	5.0	1	1.4	
> 12 months	4	2.8	5	7.1	

^a^ Wilcoxon's test

^b^ Fisher's test

^c^ Hypertension was defined as systolic blood pressure ≥140 and / or diastolic blood pressure ≥90 mmHg, or self-reported hypertension (referred), when indigenous reported having the diagnosis of hypertension done by a physician or a nurse or when a person take anti-hypertensive medicine, independently of the blood pressure values measured in the interview.

SD: Standard Deviation.

The Table 4 presents the results of the multivariate analysis with the odds ratio (OR) of the crude and adjusted analysis. There was a positive and statistically significant association (P <0.05) between alcohol sex. Risk of alcohol abuse was 10 times higher among men than among women (OR = 10.27, 95% CI: 5.76–18.30); living in rural areas almost 10 times (OR = 9.77, 95% CI: 5.08–18.79); and 4.72 times more for smoking compared to non-smoking. Negative and significant association occurred with age, given that as the younger age higher was the prevalence of risk for alcohol consumption. No personal history of dyslipidemia was also associated with a lower risk of alcohol abuse (OR = 0.41, 95% CI: 0.19–0.89). Referred hypertension, physical activity and BMI were maintained for adjustment of the model.

**Table 4:** Unadjusted and adjusted odds ratio (OR) of factors associated with alcohol abuse in the indigenous Mura, Brazil.

Variables	OR not adjusted (CI 95%)	P-value	OR adjusted (CI 95%)	P-value
**Sex**				
Female	1		1	
Male	6.59 (4.34–10.00)	<0.001	10.27 (5.76–18.30)	<0.001
**Age**	0.98 (0.96–0.99)	0.001	0.95 (0.94–0.97)	<0.001
**Living area**				
Rural	4.70 (3.12–7.08)	<0.001	9.77 (5.08–18.79)	<0.001
Urban	1		1	
**Smoker**				
Yes	3.49 (2.12–5.74)	<0.001	4.72 (2.35–9.46)	<0.001
No	1		1	
Ex-smoker	1.40 (0.88–2.23)	0.149	1.55 (0.84–2.86)	0.104
**Referred Hypertension**				
Yes	1		1	
No	1.93 (1.17–3.18)	0.010	0.97 (0.48–1.92)	0.928
**Personal history of Dyslipidemia**				
Yes	1		1	
No	1.48 (0.81–2.70)	0.191	0.41 (0.19–0.89)	0.024
Do not Know	3.84 (2.00–7.35)	<0.001	0.84 (0.36–1.96)	0.690
Body Mass Index (BMI)	0.97 (0.93–1.01)		1.04 (0.99–1.10)	0.100
Physical activity (IPAQ)				
Active	1		1	
Sedentary	0.91 (0.62–1.32)	0.629	0.88 (0.54–1.42)	0.604

CI: Confidence Interval; IPAQ = International Physical Activity Questionnaire

Model adjusted for: sex, age, living area (urban or rural), smoker (yes, no or ex-smoker), referred hypertension (yes or no), personal history of dyslipidemia (yes, no or do not know), body mass index (BMI), physical activity (active, sedentary).

## Discussion

In this study, the association between alcohol and hypertension was evidenced only for referred hypertension. However, we consider it a relevant problem, since often when people receive a diagnosis of disease, they are directed to adopt healthy lifestyles, among them, the exclusion of alcohol consumption. Further studies are needed to corroborate this hypothesis. However, both events when analyzed separately are relevant because they are chronic problems affecting the indigenous population, which would not be expected to suffer from such problems.

The sample of indigenous Mura studied was predominantly composed of young adults of productive age, revealing a different demographic profile of the non-indigenous population. The apparent predominance of women in the sample (57.8%) may indicate the demographic change, characterized by the temporary exit of the man from the village to work in the city. Data from the 2010 Demographic Census of the Brazilian Institute of Geography and Statistics (IBGE) [31] showed a balance between the sexes in the indigenous population and the analysis of the sex ratio by household situation, a behavior similar to that of the non-indigenous population was observed, with a predominance of women urban areas and men in rural areas. It is noteworthy in the present study that the male sex has a chance of 10 times of risk for alcohol consumption in the indigenous Mura. In one of the first studies that evaluated the alcoholism in indigenous of Terena's villages in the Center-West Region of Brazil also the indices were higher in the men. In the indigenous aged over 15 years the prevalence of alcoholic beverage was 31.0% in males and 1.6% in females [32]. Four years later, another study with the same ethnic group showed a rise in women to 17.1%, while in men there was a decrease (22.4%), but in this study the sample was indigenous living on the outskirts or fringe of the city, while in the previous study the indigenous lived in villages [33]. In relation to sex, the amount of alcoholic beverage has different characteristics. A study with a non-indigenous population confirmed a protective action for women consuming up to 15g of ethanol/day (OR = 0.49) and deleterious for men consuming more than 30g of ethanol/day (OR = 2.94) [34].

The demographic factors included in this analysis, especially in the multivariate regression, are used precisely to adjust for these factors, which are associated with both alcohol consumption and hypertension. We therefore consider it appropriate to include such variables. In addition, we emphasize that this is the first study that deals with this issue in indigenous, and therefore, serves as an exploratory approximation of the factors that may be associated with alcohol consumption, since the population profile itself may be a factor associated with alcohol consumption.

The advancement of the age had a protective action to risk for alcohol abuse in the indigenous Mura confirming that the younger ones were the ones that had higher prevalence of risk for alcohol abuse, therefore, can suffer early to the harmful actions of alcohol. We also highlight that in multivariate analysis, the variable age and sex changed the significance of referred hypertension. Maybe because older people have higher prevalence of hypertension. In opposite the young that have higher risk for alcohol abuse. Higher prevalence of hypertension in older people is observed in a population-based cross-sectional survey conducted in Tanzania where the association between alcohol and hypertension is analyzed[35].

It is noteworthy that among the interviewed sample with risk for alcohol abuse, more than half resided in the rural area. Maybe living in the rural area contributed for adjusted odds ratio = 9.77, so almost 10 times higher compared to those who live in urban area. This fact can be related to the coexistence in community and the lack of leisure options, causing the population to gather around the alcoholic beverage. The presence of alcohol and its consumption in the indigenous festivities reveals that the drink is related to the integration of individuals in the community [36].

In the family constitution of the indigenous Mura, it was observed that the majority of participants who consumed alcoholic beverages lived with a partner and had about three children. They are large families when compared to the current Brazilian population, because according to the IBGE demographic census of 2010, the average was 1.9 children by family [37]. This profile of large families of indigenous people, associated with financial difficulty, enhances social vulnerability, favoring alcoholism.

As indigenous education and income are low, thus reflects in the low socioeconomic level. It is noteworthy that in the inland of the Amazon there is precariousness and difficulty of access to education, in addition to unsatisfactory conditions for indigenous people to provide for themselves and the family. This poverty can be observed by the data that 97.7% are beneficiary of government income transfer programme, called Bolsa Família (Family Grant). Such conditions may be a potential risk to both the health of the individual and their family members, which, when associated with the damages caused by the high consumption of the alcoholic beverage, leads to its abusive use.

Fishing and sale of agricultural production and crafts are resources used to supplement income in the indigenous population and in the present study these activities were associated with risk for alcohol abuse (P <0.05) and such finding was also demonstrated in a study with indigenous Bororo of Mato Grosso, but situations with an excess of alcoholic beverages and "drunks"—very common among them—and interfered in the continuity of this type of job, since they stop to working in order to alcohol drinking and also spend money with this [38].

Regarding the habits and lifestyles of the indigenous Mura, smoking was a variable that remained in the model after adjustments (OR = 4.72), indicating that who have risk for alcohol abuse have also higher prevalence of smoking compared to those that do no smoke. The percentage of smokers among the indigenous with risk for alcohol abuse was 31.7%, therefore, higher than the general population in Brazil, which in 2013 was 14.7%. In indigenous people of the Jaguapiru village in the city of Dourados/MT, smoking prevalence was 19.0% [13]. Therefore, this is a worrying situation since cigarette smoking is also considered a major risk factor for cardiovascular disease [39].

As to the personal history, an inverse association (P <0.05) was found between risk for alcohol consumption and history of dyslipidemia, cardiac problems and diabetes mellitus, as well as total cholesterol levels. However, only not have antecedents for dyslipidemia remained in the model after the adjustments (OR = 0.41). These findings suggest that the presence of comorbidities increases the commitment to health and avoid behaviors that can further aggravate the health condition. Studies have shown that moderate alcohol consumption may result in higher rates of HDL-cholesterol and low C-reactive protein that minimize the likelihood of aggravations, such as dyslipidemia [4] and decreased cardiovascular risk [39].

The evaluation of the anthropometric characteristics of the indigenous identified a positive association (P <0.05) between risk for alcohol consumption and neck circumference, skeletal muscle ratio, weight and height. Although these variables were not maintained in the final adjusted model, the importance of these variables in the context of cardiovascular risk factors is emphasized. In addition, high levels of central adiposity (23.2%) were found in the total indigenous Mura studied. More than half of the indigenous were obese or overweight, a fact already shown in other studies [40, 41], and alcohol consumption is directly related to weight gain, even becoming overweight and obese [42], since this behavior is result of changes in the life style and in the indigenous culture, as identified in this research.

Indigenous food intake was also studied and an association between risk for alcohol abuse and higher score for consumption of processed products (P <0.05), it was also observed that higher *in natura* consumption of food are associated to no-risk for alcohol consumption but not included in the final adjustment model. Although we must worry about the habit for industrialized food consumption, as it is one practice when people drink, because they use as appetizers, which are frequently fat and processed foods. In this sense, it is important to maintain traditional habits such as hunting, fishing and food cultivation, to keep a natural food consumption and preserve the indigenous original culture. On the other hand, the consumption of industrialized foodstuffs, characterized by the nutritional transition in which indigenous peoples began to adopt habits and lifestyles of non-indigenous populations, was also identified in the indigenous population.

Regarding the distribution of indigenous Mura according to blood pressure levels, it was verified that they remained within the normal range [21] and there was no difference in the prevalence of hypertension between those with risk (23%) and no-risk for alcohol abuse (29%). However, the referred hypertension is associated to no-risk for problematic alcohol consumption (14.2% *vs* 24.3%, P = 0.009). We emphasize that this is the first study that seeks to identify the association between alcohol consumption and hypertension in indigenous people. Our effort was to illuminate this topic, which is still incipient, and even if we found association between hypertension and alcohol consumption in the bivariate analysis, it was not significant in the multivariate analysis, but it was maintained in the final model, with BMI and physical activity, in order to the model adjust, considering that the latter two may be confounders in the association between alcohol and hypertension[43, 44]. Our interpretation is that the change in the way of life of the indigenous approaching the way of life of non-indigenous, especially due to urbanization, also increases the prevalence of both problems, which can be detected by the association between the housing zone (Urban / rural) and alcohol consumption. Another explanation is the relationship between hypertension and sex and age, because women have higher prevalence of hypertension and lower prevalence of alcohol abuse; and younger people have higher prevalence of alcohol abuse and lower prevalence of hypertension.

The present study limited itself to analyzing the characteristics of indigenous in a timely manner, an inherent aspect of cross-sectional design, in which factor and outcome evaluations do not concomitantly establish cause and effect relationships. On the other hand, an instrument was adopted, worldwide, in the evaluation of alcoholic beverage intake, minimizing bias in the identification of this data.

The prevalence of alcohol abuse and hypertension identified in the present study were high. The findings point to the importance of establishing health policies with a focus on the prevention of cardiovascular risk factors with health education strategies for the adoption of healthy habits and lifestyles. Thus, the multidisciplinary work of the health team in the attention to the natives is necessary in order to meet the real needs of this population and to modify the profile of morbimortality resulting from the epidemiological transition that these peoples experience.

The Mura indigenous population presents cultural changes, when adopting the urban way of life, especially when it is considered that they are speakers of the Portuguese language, which denotes the proximity of coexistence in urban areas and with non-indigenous ones. This was due to proximity and ease of access and communication with urban areas, as well as access to previously unavailable goods and services. With this, we see a population that no longer lives as village natives, or as those inhabitants of rural areas. Thus, they are also exposing themselves to the characteristic risk factors of the urbanization process. Perhaps this is responsible for the prevalence of alcoholism and hypertension detected in the study.

## Supporting information

S1 Appendix "caderno de entrevista.docx": Questionnaire used in this field research. (DOCX)

## References

[1] Coimbra CEA Jr, Santos RV. [Health, minorities and inequality: some webs of inter-relations, emphasizing indigenous peoples in Brazil]. Ciênc saúde coletiva. 2000;5(1):125–32. 10.1590/S1413-81232000000100011.

[2] Souza ML, Garnelo L. ["It sure ain't easy!": an ethnographic study of primary health care for patients with hypertension and/or diabetes in Manaus, Amazonas State, Brazil]. Cadernos de saude publica. 2008;24 (Suppl 1):S91–9. 10.1590/S0102-311X2008001300014. . 18660917

[3] Guimarães LAM, Grubits S. [Alcoholism and violence in indigenous ethnic groups: a critical view of the brazilian situation]. Psicol Soc. 2007;19(1):45–51. 10.1590/S0102-71822007000100007.

[4] Almeida TSO, Fook SML, Mariz SR. [Association between alcoholism and subsequente hypertension: a systematic review]. RSC online [Internet]. 2016 [cited 2016 Nov 16]; 5(1):[76–90 pp.]. Available from: http://www.ufcg.edu.br/revistasaudeeciencia/index.php/RSC-UFCG/article/view/328/229.

[5] Mancilha-Carvalho JJ, Carvalho JV, Lima JA, Sousa e Silva NA. [The absence of risk factors for coronary disease in Yanomami Indians and the influence of acculturation on arterial pressure]. Arq Bras Cardiol. 1992;59(4):275–83. 1341184

[6] Mancilha-Carvalho JJ, Oliveira R, Esposito RJ. Blood pressure and electrolyte excretion in the Yanomamo Indians, an isolated population. Journal of human hypertension. 1989;3(5):309–14. Epub 1989/10/01. . 2810327

[7] Mancilha-Carvalho JJ, Sousa e Silva NA, Carvalho JV, Lima JA. [Blood pressure in 6 Yanomami villages]. Arq Bras Cardiol. 1991;56(6):477–82. 1823749

[8] Mancilha-Carvalho JJ, Souza e Silva NA. The Yanomami Indians in the INTERSALT Study. Arq Bras Cardiol. 2003;80(3):289–300. 12856272 10.1590/s0066-782x2003000300005

[9] Oliver WJ, Cohen EL, Neel JV. Blood pressure, sodium intake, and sodium related hormones in the Yanomamo Indians, a "no-salt" culture. Circulation. 1975;52(1):146–51. Epub 1975/07/01. . 1132118 10.1161/01.cir.52.1.146

[10] Souza Filho ZA, Ferreira AA, Santos BD, Pierin AM. [Hypertension prevalence among indigenous populations in Brazil: a systematic review with meta-analysis]. Revista da Escola de Enfermagem da U S P. 2015;49(6):1016–26. Epub 2016/07/16. 10.1590/S0080-623420150000600019 . 27419687

[11] [II National survey about alcohol and drugs (LENAD)]. São Paulo: Instituto Nacional de Ciência e Tecnologia para Políticas Públicas de Álcool e Outras Drogas (INPAD), UNIFESP; 2014 [cited 2016 Nov 16]. http://inpad.org.br/wp-content/uploads/2014/03/Lenad-II-Relat%C3%B3rio.pdf.

[12] Melo JRF, Maciel SC, Oliveira RCC, Silva AO. [Implications of alcohol abuse and consumption in the Potiguara indigenous community]. Physis. 2011;21(1):319–33. 10.1590/S0103-73312011000100019.

[13] Oliveira GF, Oliveira TR, Ikejiri AT, Andraus MP, Galvao TF, Silva MT, et al Prevalence of hypertension and associated factors in an indigenous community of central Brazil: a population-based study. PloS one. 2014;9(1):e86278 Epub 2014/02/04. 10.1371/journal.pone.0086278 ; 24489710 PMC3904906

[14] Souza MLP, Deslandes SF, Garnelo L. [Ways of life and ways to drink of young indigenous in a transformation context]. Ciênc saúde coletiva. 2010;15(3):709–16. 10.1590/S1413-81232010000300013. 20464183

[15] Cardoso A, Coimbra Júnior C, Santos RV. [National Survey of Health and Nutrition of Indigenous Peoples, 1st, 2010]: Consórcio ABRASCO (Associação Brasileira de Pós- Graduação em Saúde) & Institute of Ibero-American Studies, Goteborg University, Suécia; 2010 [cited 2016 Nov 16]. http://brasil.campusvirtualsp.org/node/181972.

[16] ABIA. [Scenario of sodium consumption in Brazil: study elaborated based on Brazilian Institute of Geography and Statistics] São Paulo: Associação Brasileira das Indústrias de Alimentação (ABIA); 2013 [cited 2017 Feb 26]. http://www.abia.org.br/sodio/Sodio2.pdf.

[17] Brasil. Ministério da Saúde. VIGITEL BRASIL 2014 - [Surveillance of risk factors and protection for cronic diseases using telephone survey]. Brasília: Ministério da Saúde, Secretaria de Vigilância em Saúde. Departamento de Vigilância de Doenças e Agravos não Transmissíveis e Promoção da Saúde.; 2015.

[18] WHO. World Health Organization. Problems related to alcohol consumption Report of a WHO Expert Committee.Technical Report. Series 650. Geneva: World Health Organization; 1980 [cited 2016 Nov 16]. http://whqlibdoc.who.int/trs/WHO_TRS_650.pdf?ua=1. 6777993

[19] Vera-Cala LM, Orostegui M, Valencia-Angel LI, López N, Bautista LE. Accuracy of the Omron HEM-705 CP for blood pressure measurement in large epidemiologic studies. Arq Bras Cardiol. 2011;96(5):393–8. 10.1590/S0066-782X2011005000038. 21468531

[20] James PA, Oparil S, Carter BL, Cushman WC, Dennison-Himmelfarb C, Handler J, et al 2014 evidence-based guideline for the management of high blood pressure in adults: report from the panel members appointed to the Eighth Joint National Committee (JNC 8). Jama. 2014;311(5):507–20. Epub 2013/12/20. 10.1001/jama.2013.284427 . 24352797

[21] Malachias M, Souza V, Plavnik F, Brandao A, Neves M, Bortolotto L, et al VII Brazilian Guidelines on Hypertension. Arq Bras Cardiol. 2016;107(supl.3):1–83. 10.5935/abc.20160151.

[22] WHO. World Health Organization. Obesity: preventing and managing the global epidemic. Geneve: World Health Organization; 2000 [cited 2016 Nov 16]. Obesity: preventing and managing the global epidemic WHO. 11234459

[23] Tibana RA, Teixeira TG, Farias DL, Silva Ade O, Madrid B, Vieira A, et al Relation of neck circumference and relative muscle strength and cardiovascular risk factors in sedentary women. Einstein. 2012;10(3):329–34. Epub 2013/02/07. . 23386013 10.1590/s1679-45082012000300013

[24] Ben-Noun L, Sohar E, Laor A. Neck circumference as a simple screening measure for identifying overweight and obese patients. Obesity research. 2001;9(8):470–7. 10.1038/oby.2001.61 11500527

[25] Chan DC, Watts GF, Barrett PH, Burke V. Waist circumference, waist-to-hip ratio and body mass index as predictors of adipose tissue compartments in men. QJM: monthly journal of the Association of Physicians. 2003;96(6):441–7. Epub 2003/06/06. . 12788963 10.1093/qjmed/hcg069

[26] [Instruction Manual for Body Control Scale (Bioimpedance Scale) Model HBF-514C] 2014 [cited 2016 Nov 16]. http://www.omronbrasil.com/uploads/attachment/180e655c39164512d2ba7abbd8f70cefd75e6083HBF-514C-pdf.pdf.

[27] Pitanga FJG, Lessa I. [Sensitivity and specificity of the conicity index as a coronary risk predictor among adults in Salvador, Brazil]. Rev bras epidemiol. 2004;7:259–69. 10.1590/S1415-790X2004000300004.

[28] Sociedade Brasileira de Diabetes. [Guidelines of the Brazilian Diabetes Society: 2014–2015]. São Paulo: AC Farmaceutica; 2015 [cited 2016 Nov 3]. https://www.diabetes.org.br/images/2015/area-restrita/diretrizes-sbd-2015.pdf.

[29] Xavier HT, Izar MC, Faria Neto JR, Assad MH, Rocha VZ, Sposito AC, et al [V Brazilian Guideline on Dyslipidemias and Prevention of Atherosclerosis]. Arq Bras Cardiol. 2013;101(4. Supl.1):1–20. 10.5935/abc.2013S010. 24217493

[30] IPAQ. Research Committee. International physical activity questionnaire. [June 30, 2017]. https://sites.google.com/site/theipaq/scoring-protocol.

[31] IBGE. Brazilian Institute of Geography and Statistics. [Indigenous Brazil: Sociodemographic and domiciliary characteristics] 2016 [cited 2016 Oct 04]. http://indigenas.ibge.gov.br/estudos-especiais-3/o-brasil-indigena/caracteristica-socidemograficas-e-domiciliares.

[32] Albuquerque J, Souza JA. Prevalence of alcoholism in Terena indigenous population of Sidrolândia Complex—Colonia Dois Irmãos. Anais da I oficina Macro regional de Estratégia, Prevenção e Controle das DST/AIDS para populações indígenas das regiões Sul, Sudeste e do Mato Grosso do Sul. Brasília: Ministério da Saúde; 1997.

[33] Aguiar JI, Souza JA. Alcoholism in Terena indigenous population of Mato Grosso do Sul state—impact of surrounding society. Anais do seminário sobre alcoolismo e vulnerabilidade ás DST/AIDS entre os povos indígenas da magrorregião sul, sudeste e mato Grosso do sul N ° 4. Brasília: Secretaria de Políticas de Saúde. Ministério da Saúde; 1997.

[34] Martins MSAS, Ferreira MG, Guimarães LV, Vianna LAC. Hypertension and lifestyle in Sinop, a Municipality in the Legal Amazon Region. Arq Bras Cardiol. 2010;94(5):639–44. 10.1590/S0066-782X2010005000028. 20428725

[35] Kavishe B, Biraro S, Baisley K, Vanobberghen F, Kapiga S, Munderi P, et al High prevalence of hypertension and of risk factors for non-communicable diseases (NCDs): a population based cross-sectional survey of NCDS and HIV infection in Northwestern Tanzania and Southern Uganda. BMC Medicine. 2015;13:126 10.1186/s12916-015-0357-9 26021319 PMC4476208

[36] Ghiggi A Junior, Langdon EJ. Reflections on intervention strategies with respect to the process of alcoholization and self-care practices among Kaingang indigenous people in Santa Catarina State, Brazil. Cadernos de saude publica. 2014;30(6):1250–8. Epub 2014/08/08. . 25099048 10.1590/0102-311x00108613

[37] IBGE. Brazilian Institute of Geography and Statistics. [Census 2010. Indigenous general characteristics—Results of Universe] 2010 [cited 2016 Oct 04]. http://www.ibge.gov.br.

[38] Viertler R. [Inter-ethnic conviviality and alcoholism among the Bororo: results of a research]. Revista Tellus [Internet]. 2002 [cited 2016 Oct 06]; 2(2):[9–38 pp.]. Available from: http://www.tellus.ucdb.br/projetos/tellus/index.php/tellus/article/view/8/8.

[39] Higashiyama A, Okamura T, Watanabe M, Kokubo Y, Wakabayashi I, Okayama A, et al Alcohol consumption and cardiovascular disease incidence in men with and without hypertension: the Suita study. Hypertension research: official journal of the Japanese Society of Hypertension. 2013;36(1):58–64. Epub 2012/08/31. 10.1038/hr.2012.133 . 22932877

[40] Favaro TR, Santos RV, Cunha GM, Leite Ida C, Coimbra CE Jr. [Obesity and overweight in adult Xukuru of Ororuba Indians, Pernambuco State, Brazil: magnitude and associated socioeconomic and demographic factors]. Cadernos de saude publica. 2015;31(8):1685–97. Epub 2015/09/17. 10.1590/0102-311X00086014 . 26375647

[41] Lourenco AE, Santos RV, Orellana JD, Coimbra CE Jr. Nutrition transition in Amazonia: obesity and socioeconomic change in the Surui Indians from Brazil. American journal of human biology: the official journal of the Human Biology Council. 2008;20(5):564–71. Epub 2008/04/30. 10.1002/ajhb.20781 . 18442078

[42] Cibeira GH, Muller C, Lazzaretti R, Nader GA, Caleffi M. [Alcohol consumption, social and economic factors and excess weight: a cross-sectional study]. Cien Saude Colet. 2013;18(12):3577–84. Epub 2013/11/23. . 24263874 10.1590/s1413-81232013001200014

[43] Gruber S, van der Laan MJ. Consistent causal effect estimation under dual misspecification and implications for confounder selection procedures. Statistical methods in medical research. 2015;24(6):1003–8. Epub 2012/03/01. 10.1177/0962280212437451 . 22368176 PMC4081493

[44] Jardim PCBV, Gondim MRP, Monego ET, Moreira HG, Vitorino PVO, Souza WKSB, et al Hipertensão arterial e alguns fatores de risco em uma capital brasileira. Arq Bras Cardiol. 2007;88:452–7. 17546277

