# Supplementary material for: Relationship between alcohol drinking and arterial hypertension in indigenous people of the Mura ethnics, Brazil
Source: PLoS One. 2017 Aug 4;12(8):e0182352. doi: 10.1371/journal.pone.0182352 (PMC5544198; doi:10.1371/journal.pone.0182352)
Supplement: S1 Appendix — (DOCX) [file pone.0182352.s001.docx]

**“Avaliação de fatores de risco cardiovascular, com ênfase na hipertensão arterial, em indígenas da etnia mura: estudo comparativo entre população rural e urbana.”**

| **Entrevista**  **Data**  **Horário de início :**  **Horário de término :** | **Revisão e Checagem**  **Data**  **Horário de início :**  **Horário de término :** |
| --- | --- |

**Nome:**

**Sexo: Masculino Feminino**

**Data de Nascimento: Idade:** (Só se não souber a data de nascimento)

**Zona: Urbana Rural**

**Telefone:**

**Caso a entrevista não tenha sido realizada, marque a opção que corresponde ao motivo da sua não realização:**

**Recusa em assinar o TCLE Recusa à entrevista Não se aplica**

**Outro:___________________________**


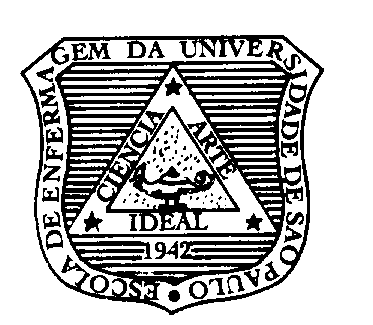


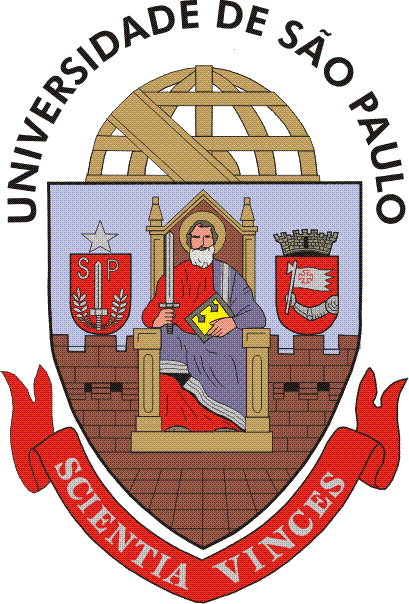


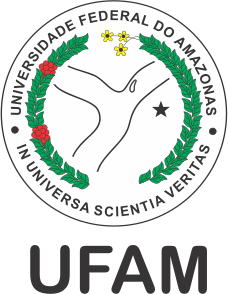


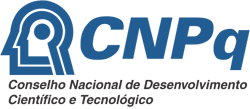


**Ficha de Exame Físico**

**1. Avaliação Antropométrica**

1.1 Peso:____________kg 1.2 Altura: ____________m

1.3 Bioimpedância:____________% gordura 1.4 Circunferência do pescoço:________cm

1.5 Circunferência da Cintura:_________ cm 1.6 Circunferência do Quadril:_________cm

1.7 Relação Cintura Quadril:__________cm

**2. Medida da Pressão Arterial**

*2.1 Primeira Medida*

Sistólica: ______________ mmHg Diastólica: ______________ mmHg

*2.2 Segunda Medida*

Sistólica: ______________ mmHg Diastólica: ______________ mmHg

*2.3 Terceira Medida*

Sistólica: ______________ mmHg Diastólica: ______________ mmHg

**3. Medida da Glicemia Capilar**

3.1 Glicose em Jejum:____________mg/dL

**4. Medida do Perfil Lipídico**

4.1 Triglicérides:____________mg/dL

4.2 Colesterol:______________mg/dL

**Caracterização Sócio Econômica e do Domicílio**
(*Adaptado do Inquérito Nacional de Saúde e Nutrição dos Povos Indígenas CADERNO 2, http://www.abrasco.org.br/grupos/arquivos/2013-01-23%2013:44:23.pdf*)

**5. Estado Civil:**

( 1 ) Solteiro ( 2 ) Casado ( 3 ) Viúvo ( 4 ) Amasiado ( 5 ) Separado

**6. Quantos filhos você tem?**____________

**7. Qual foi o mais elevado curso escolar que você frequentou ou frequenta atualmente?**

( 1 ) Nenhum ( 2 ) Regular do ensino fundamental

( 3 ) Educação de jovens e adultos ou supletivo do ensino fundamental – séries iniciais

( ) (1⁰ ao 5⁰ ano) ( ) (6⁰ ao 9⁰ ano)

( 4 ) Regular do ensino fundamental ( ) ou médio ( )

( 5 ) Superior de graduação. Qual? _________________

( 6 ) Pós-graduação

**8. Qual a sua Renda Mensal?**_________________

**9. Qual a Renda Mensal da sua Família?**________

**10. Quantas Pessoas Dependem da Renda Familiar Mensal?**___________________

**11. Quem é o Responsável pela Renda Familiar (Chefe da Família)?**

( 1 ) Participante ( 2 ) Cônjuge ( 3 ) Casal ( 4 ) Filho ( 5 ) Não sabe informar

( 6 ) Outro _________________

**12. No último ano (12 meses), o dinheiro que os moradores do domicílio usam veio de:**

**Sim**  **Não**

12.1. Trabalho remunerado – ano todo 1 2

12.2. Trabalho remunerado – temporário 1 2

12.3. Venda de produtos da agricultura/pecuária/pesca 1 2

12.4. Venda de artesanato ou produção cultural 1 2

12.5. Aposentadoria 1 2

12.6. Benefícios sociais 1 2

12.7. Outro - Especificar:_____________________________________

**13. Caracterização física do domicílio** (MARQUE O TIPO PREDOMINANTE)

13.1 Tipo de piso:

( 1 ) Chão de terra ( 2 ) Madeira ( 3 ) Cerâmica ( 4 ) Cimento ( 5 ) Flutuante

13.2 Tipo de parede:

( 1 ) Palha ( 2 ) Madeira ( 3 ) Tijolo ( 4 ) Taipa/barro ( 5 ) Lona/plástico

13.3 Tipo de cobertura/telhado:

( 1 ) Palha ( 2 ) Madeira ( 3 ) Laje ( 4 ) Lona/plástico ( 5 ) Telha de barro ( 6 ) Telha de zinco ou amianto

**14. Caracterização socioeconômica do domicílio**

14.1 Rádio AM/FM ( 1 ) Sim Quantos______ ( 2 ) Não

14.2 Televisão ( 1 ) Sim Quantos______ ( 2 ) Não

14.3 Aparelho de DVD ( 1 ) Sim Quantos______ ( 2 ) Não

14.4 Geladeira e/ou freezer ( 1 ) Sim Quantos______ ( 2 ) Não

14.5 Fogão ( 1 ) Sim Quantos______ ( 2 ) Não

14.6 Forno de microondas ( 1 ) Sim Quantos______ ( 2 ) Não

14.7 Máquina de lavar roupa ( 1 ) Sim Quantos______ ( 2 ) Não

14.8 Telefone celular ( 1 ) Sim Quantos______ ( 2 ) Não

14.9 Linha de telefone fixo ( 1 ) Sim Quantos______ ( 2 ) Não

14.10 Rede internet ( 1 ) Sim Quantos______ ( 2 ) Não

14.11 Computador / Noteboook / Tablet ( 1 ) Sim Quantos______ ( 2 ) Não

14.12 Aparelho de ar-condicionado ( 1 ) Sim Quantos______ ( 2 ) Não

14.13 Antena parabólica ( 1 ) Sim Quantos______ ( 2 ) Não

14.14 Motoserra ( 1 ) Sim Quantos______ ( 2 ) Não

14.15 Motor de popa/ Rabeta ( 1 ) Sim Quantos______ ( 2 ) Não

14.16 Animal de carga/trabalho

(cavalo / burro / jumento / boi) ( 1 ) Sim Quantos______ ( 2 ) Não

14.17 Ralador de mandioca com motor ( 1 ) Sim Quantos______ ( 2 ) Não

14.18 Bicicleta ( 1 ) Sim Quantos______ ( 2 ) Não

14.19 Motocicleta ( 1 ) Sim Quantos______ ( 2 ) Não

14.20 Automóvel/Carro ( 1 ) Sim Quantos______ ( 2 ) Não

**15. Número de moradores no domicílio:**

| 15.1 Morador | 15.2 Sexo | 15.3 Idade |
| --- | --- | --- |
| 1) | ( 1 ) Fem ( 2 ) Masc |  |
| 2) | ( 1 ) Fem ( 2 ) Masc |  |
| 3) | ( 1 ) Fem ( 2 ) Masc |  |
| 4) | ( 1 ) Fem ( 2 ) Masc |  |
| 5) | ( 1 ) Fem ( 2 ) Masc |  |
| 6) | ( 1 ) Fem ( 2 ) Masc |  |
| 7) | ( 1 ) Fem ( 2 ) Masc |  |
| 8) | ( 1 ) Fem ( 2 ) Masc |  |
| 9) | ( 1 ) Fem ( 2 ) Masc |  |
| 10) | ( 1 ) Fem ( 2 ) Masc |  |

**Critérios de Classificação Econômica**

No Domicílio têm?

|  |  | **Quantidade que possui** | | | |
| --- | --- | --- | --- | --- | --- |
| **Itens de Conforto** | **Não**  **possui** | **1** | **2** | **3** | **4 +** |
| Quantidade de automóveis de passeio exclusivamente para uso particular |  |  |  |  |  |
| Quantidade de empregados mensalistas, considerando apenas os que trabalham pelo menos cinco dias por semana |  |  |  |  |  |
| Quantidade de máquinas de lavar roupa, excluindo tanquinho |  |  |  |  |  |
| Quantidade de banheiros |  |  |  |  |  |
| DVD, incluindo qualquer dispositivo que leia DVD e considerando DVD de automóvel |  |  |  |  |  |
| Quantidade de geladeiras |  |  |  |  |  |
| Quantidade de *freezers* independentes ou parte da geladeira duplex |  |  |  |  |  |
| Quantidade de microcomputadores, considerando computadores de mesa, laptops, notebooks e netbooks e considerando tablets, palms ou smartphones |  |  |  |  |  |
| Quantidade de lavadora de louças |  |  |  |  |  |
| Quantidade de fornos de micro-ondas |  |  |  |  |  |
| Quantidade de motocicletas, desconsiderando as usadas exclusivamente para uso profissional |  |  |  |  |  |
| Quantidade de máquinas secadoras de roupas, considerando lava e seca |  |  |  |  |  |

| **A água utilizada neste domicílio é proveniente de?** | |
| --- | --- |
| 1 | Rede geral de distribuição |
| 2 | Poço ou nascente |
| 3 | Outro meio |

| **Considerando o trecho da rua do seu domicílio, você diria que a rua é:** | |
| --- | --- |
| 1 | Asfaltada / Pavimentada |
| 2 | Terra / Cascalho |

Sistemas de Pontos

Variavéis

|  | **Quantidade** | | | | |
| --- | --- | --- | --- | --- | --- |
|  | **0** | **1** | **2** | **3** | **4 ou +** |
| Banheiros | 0 | 3 | 7 | 10 | 14 |
| Empregados domésticos | 0 | 3 | 7 | 10 | 13 |
| Automóveis | 0 | 3 | 5 | 8 | 11 |
| Microcomputador | 0 | 3 | 6 | 8 | 11 |
| Lava louça | 0 | 3 | 6 | 6 | 6 |
| Geladeira | 0 | 2 | 3 | 5 | 5 |
| Freezer | 0 | 2 | 4 | 6 | 6 |
| Lava roupa | 0 | 2 | 4 | 6 | 6 |
| DVD | 0 | 1 | 3 | 4 | 6 |
| Micro-ondas | 0 | 2 | 4 | 4 | 4 |
| Motocicleta | 0 | 1 | 3 | 3 | 3 |
| Secadora roupa | 0 | 2 | 2 | 2 | 2 |

Grau de instrução do chefe de família e acesso a serviços públicos

| **Escolaridade da pessoa de referência** | | |
| --- | --- | --- |
| Analfabeto / Fundamental I incompleto | | 0 |
| Fundamental I completo / Fundamental II incompleto | | 1 |
| Fundamental II completo / Médio incompleto | | 2 |
| Médio completo / Superior incompleto | | 4 |
| Superior completo | | 7 |
|  |  |  |
| **Serviços públicos** | | |
|  | **Não** | **Sim** |
| Água encanada | 0 | 4 |
| Rua pavimentada | 0 | 2 |

Qual é o grau de instrução do chefe da família? Considere como chefe da família a pessoa que contribui com a maior parte da renda do domicílio.

| **Nomenclatura atual** | **Nomenclatura anterior** |
| --- | --- |
| Analfabeto / Fundamental I incompleto | Analfabeto / Primário incompleto |
| Fundamental I completo / Fundamental II incompleto | Primário completo / Ginásio incompleto |
| Fundamental completo / Médio incompleto | Ginásio completo / Colegial incompleto |
| Médio completo / Superior incompleto | Colegial completo / Superior incompleto |
| Superior completo | Superior completo |

Corte do Critério Brasil

| **Classe** | **Pontos** |
| --- | --- |
| A | 45 – 100 |
| B1 | 38 – 44 |
| B2 | 29 – 37 |
| C1 | 23 – 28 |
| C2 | 17 – 22 |
| D-E | 0 – 16 |

| **Avaliação dos Hábitos Alimentares dos Indígenas Mura** *(Adaptado do Inquérito Nacional de Saúde e Nutrição dos Povos Indígenas CADERNO 2, http://www.abrasco.org.br/grupos/arquivos/2013-01-23%2013:44:23.pdf)* *e no Instrumento utilizado na Vigilância de Doenças Crônicas por Inquérito Telefônico - VIGITEL* | | | | | | | | | | | | | | | | |  |  |  |  |  |  |  |  |  |  |  |  |  |  |  |  |  |  |  |  |  |  |  |  |  |  |  |  |  |  |  |  |  |  |  |  |  |  |  |  |  |  |  |  |  |  |  |  |  |  |  |  |  |  |  |  |  |  |
| --- | --- | --- | --- | --- | --- | --- | --- | --- | --- | --- | --- | --- | --- | --- | --- | --- | --- | --- | --- | --- | --- | --- | --- | --- | --- | --- | --- | --- | --- | --- | --- | --- | --- | --- | --- | --- | --- | --- | --- | --- | --- | --- | --- | --- | --- | --- | --- | --- | --- | --- | --- | --- | --- | --- | --- | --- | --- | --- | --- | --- | --- | --- | --- | --- | --- | --- | --- | --- | --- | --- | --- | --- | --- | --- |
|  | | | | | | | | | | | | | | | | |  |  |  |  |  |  |  |  |  |  |  |  |  |  |  |  |  |  |  |  |  |  |  |  |  |  |  |  |  |  |  |  |  |  |  |  |  |  |  |  |  |  |  |  |  |  |  |  |  |  |  |  |  |  |  |  |  |  |
| **16. Os alimentos que os moradores do domicílio consomem vêm de:**  (ASSINALAR AS ALTERNATIVAS RELATADAS ESPONTANEAMENTE PELO ENTREVISTADO). | | | | | | | Sim | Não | | | | |  |  | |  |  | | | |  | | | |  | | | | |  | | |  |  |  |  |  |  |  |  |  |  |  |  |  |  |  |  |  |  |  |  |  |  |  |  |  |  |  |  |  |  |  |  |  |  |  |  |  |  |  |  |  |  |
|  |  |  |  |  |  |  |  |  |  |  |  |  |  |  | |  |  | | | |  | | | |  | | | | |  | | |  |  |  |  |  |  |  |  |  |  |  |  |  |  |  |  |  |  |  |  |  |  |  |  |  |  |  |  |  |  |  |  |  |  |  |  |  |  |  |  |  |  |
| 16.1 Cultivo ou criação domiciliar | | | | | | | 1 | 2 | | | | |  |  | |  |  | | | |  | | | |  | | | | |  | | |  |  |  |  |  |  |  |  |  |  |  |  |  |  |  |  |  |  |  |  |  |  |  |  |  |  |  |  |  |  |  |  |  |  |  |  |  |  |  |  |  |  |
| 16.2 Cultivo ou criação coletiva (da aldeia/comunidade) | | | | | | | 1 | 2 | | | | |  |  | |  |  | | | |  | | | |  | | | | |  | | |  |  |  |  |  |  |  |  |  |  |  |  |  |  |  |  |  |  |  |  |  |  |  |  |  |  |  |  |  |  |  |  |  |  |  |  |  |  |  |  |  |  |
| 16.3 Caça ou pesca (domiciliar) | | | | | | | 1 | 2 | | | | |  |  | |  |  | | | |  | | | |  | | | | |  | | |  |  |  |  |  |  |  |  |  |  |  |  |  |  |  |  |  |  |  |  |  |  |  |  |  |  |  |  |  |  |  |  |  |  |  |  |  |  |  |  |  |  |
| 16.4 Coleta (domiciliar) | | | | | | | 1 | 2 | | | | |  |  | |  |  | | | |  | | | |  | | | | |  | | |  |  |  |  |  |  |  |  |  |  |  |  |  |  |  |  |  |  |  |  |  |  |  |  |  |  |  |  |  |  |  |  |  |  |  |  |  |  |  |  |  |  |
| 16.5 Caça, pesca ou coleta coletiva (da aldeia/comunidade) | | | | | | | 1 | 2 | | | | |  |  | |  |  | | | |  | | | |  | | | | |  | | |  |  |  |  |  |  |  |  |  |  |  |  |  |  |  |  |  |  |  |  |  |  |  |  |  |  |  |  |  |  |  |  |  |  |  |  |  |  |  |  |  |  |
| 16.6 Compra | | | | | | | 1 | 2 | | | | |  |  | |  |  | | | |  | | | |  | | | | |  | | |  |  |  |  |  |  |  |  |  |  |  |  |  |  |  |  |  |  |  |  |  |  |  |  |  |  |  |  |  |  |  |  |  |  |  |  |  |  |  |  |  |  |
| 16.7 Recebe Cesta básica | | | | | | | 1 | 2 | | | | |  |  | |  |  | | | |  | | | |  | | | | |  | | |  |  |  |  |  |  |  |  |  |  |  |  |  |  |  |  |  |  |  |  |  |  |  |  |  |  |  |  |  |  |  |  |  |  |  |  |  |  |  |  |  |  |
| 16.8 Recebe outras doações de fora (da aldeia/ comunidade) | | | | | | | 1 | 2 | | | | |  |  | |  |  | | | |  | | | |  | | | | |  | | |  |  |  |  |  |  |  |  |  |  |  |  |  |  |  |  |  |  |  |  |  |  |  |  |  |  |  |  |  |  |  |  |  |  |  |  |  |  |  |  |  |  |
| 16.9 Outros, especificar:_________________________ | | | | | | | | | | | | | | | | | | | |  | | |  | | | |  | | | | |  | | | | | | | | | | | | | | |  | | |  | | |  | | | |  | | | |  | | | | |  | |  | | |  |  |  |  |
| **17. Os moradores utilizam algum tipo de gordura no preparo dos alimentos?** ( 1 ) Sim ( 2 ) Não | | | | | | | | | | | | | | | | | | |  | | | | |  | | | | | | | | | | | | |  | | | |  | | | | |  | | | | |  | | |  | | | |  | | | |  | | |  |  |  |  |  |  |  |  |  |  |
|  |  |  |  |  |  |  |  |  |  |  |  |  |  |  |  |  |  |  |  | | | | |  | | | | | | | | | | | | |  | | | |  | | | | |  | | | | |  | | |  | | | |  | | | |  | | |  |  |  |  |  |  |  |  |  |  |
| **17.1 Sim, que tipo de gordura é utilizada?**  (ASSINALAR AS ALTERNATIVAS RELATADAS ESPONTANEAMENTE PELO ENTREVISTADO). | | | | | | | | | | | | | | | | | | |  | | | | |  | | | | | | | | | | | | |  | | | |  | | | | |  | | | | |  | | |  | | | |  | | | |  | | |  |  |  |  |  |  |  |  |  |  |
| ( 1 ) Óleo vegetal ( 2 ) Banha ou gordura | | | | | | | | | |  | | | | | | | | | | | |  | | | |  | | | | |  | | | | | | | |  | | | | |  | | | | | | | | | | | | | | |  | | | |  | | | |  | | |  | |  |  |  |
| ( 3 ) Margarina ( 4 ) Manteiga | | | | | | | | | |  | | | | | | | | | | | |  | | | |  | | | | |  | | | | | | | |  | | | | |  | | | | | | | | | | | | | | |  | | | |  |  |  |  |  | | |  | |  |  |  |
| ( 5 ) Outra, especificar:________________________________ | | | | | | | | | | | | | | | | | | | | | | | | | | | | | | | | | | | |  | |  | | | | |  | | | | |  | | | |  | | |  | | | | |  |  |  |  |  |  |  |  |  |  |  |  |  |  |  |
|  | |  | | |  | | | | |  | | | | | | | | | | | |  | | | |  | | | | |  | | | | | | | |  | | | | |  | | | | | | | | | | | | | | |  | | | |  | | | |  | | |  | |  |  |  |
| **18. Como são preparados os alimentos antes das suas refeições?** | | | | | | | | | | | | | | | | | | | | | | | | | | | | | | | | | | | |  | |  | | | | |  | | | | |  | | | |  | | |  | | | | |  |  |  |  |  |  |  |  |  |  |  |  |  |  |  |
| ( 1 ) Na maioria das vezes são fritos ( 2 ) Na maioria das vezes são fervidos e/ou cozidos | | | | | | | | | | | | | | | | | | | | | | | | | | | | | | | | | | | |  | |  | | | | |  | | | | |  | | | |  | | |  | | | | |  |  |  |  |  |  |  |  |  |  |  |  |  |  |  |
| ( 3 ) Outros. Especificar:_______________________________________ | | | | | | | | | | | | | | | | | | | | | | | | | | | | | | | | | | | |  | |  | | | | |  | | | | |  | | | |  | | |  | | | | |  |  |  |  |  |  |  |  |  |  |  |  |  |  |  |
|  | |  | | |  | | | | |  | | | | | | | | | | | |  | | | |  | | | | |  | | | | | | | |  | | | | |  | | | | | | | | | | | | | | |  | | | |  | | | |  | | |  | |  |  |  |
| **19. Os moradores costumam usar sal no preparo de alimentos?** ( 1 ) Sim ( 2 ) Não | | | | | | | | | | | | | | | | | | | | | | | | | | | | | | | | | | | |  | |  | | | | |  | | | | |  | | | |  | | |  | | | | |  |  |  |  |  |  |  |  |  |  |  |  |  |  |  |
| **19.1 Sim, que tipo de sal costumam utilizar?**  (ASSINALAR AS ALTERNATIVAS RELATADAS ESPONTANEAMENTE PELO ENTREVISTADO). | | | | | | | | | | | | | | | | | | |  | | | | |  | | | | | | | | | | | | |  | | | |  | | | | |  | | | | |  | | |  | | | |  | | | |  | | |  |  |  |  |  |  |  |  |  |  |
| ( 1 ) Sal de Cozinha ( 2 ) Sal Grosso ( 3 ) Sal Refinado | | | | | | | | | |  | | | | | | | | | | | |  | | | |  | | | | |  | | | | | | | |  | | | | |  | | | | | | | | | | | | | | |  | | | |  | | | |  | | |  | |  |  |  |
|  | | | | | | | | | |  | | | | | | | | | | | |  | | | |  | | | | |  | | | | | | | |  | | | | |  | | | | | | | | | | | | | | |  | | | |  | | | |  | | |  | |  |  |  |
| **19.2 Costumam adicionar sal nos alimentos, na hora do consumo das refeições?** ( 1 ) Sim ( 2 ) Não ou  ( 3 ) Caldo de carne em tablete ( 4 ) Caldo de galinha em tablete ( 5 ) Outros Caldos em tablete | | | | | | | | | | | | | | | | | | | | | | | | | | | | | | | | | | | |  | |  | | | | |  | | | | |  | | | |  | | |  | | | | |  |  |  |  |  |  |  |  |  |  |  |  |  |  |  |
|  |  |  |  |  |  |  |  |  |  |  |  |  |  |  |  |  |  |  |  |  |  |  |  |  |  |  |  |  |  |  |  |  |  |  |  |  | |  | | | | |  | | | | |  | | | |  | | |  | | | | |  |  |  |  |  |  |  |  |  |  |  |  |  |  |  |
| **20. Um quilo (1kg) de sal dá para quanto tempo de uso:** | | | | | | | | | | | | | | | | | | | | | | | | | | | | | | | | | | | |  | |  | | | | |  | | | | |  | | | |  | | |  | | | | |  |  |  |  |  |  |  |  |  |  |  |  |  |  |  |
| ( 1 ) Menos de um mês ( 2 ) Apenas um mês ( 3 ) Mais de um mês | | | | | | | | | | | | | | | | | | | | | | | | | | | | | | | | | | | |  | |  | | | | |  | | | | |  | | | |  | | |  | | | | |  |  |  |  |  |  |  |  |  |  |  |  |  |  |  |
| ( 4 ) Outro. Especificar:_______________________________________ | | | | | | | | | | | | | | | | | | | | | | | | | | | | | | | | | | | |  | |  | | | | |  | | | | |  | | | |  | | |  | | | | |  |  |  |  |  |  |  |  |  |  |  |  |  |  |  |
| **21. Os moradores costumam usar açúcar no preparo de alimentos?** ( 1 ) Sim ( 2 ) Não | | | | | | | | | | | | | | | | | | | | | | | | | | | | | | | | | | | |  | |  | | | | |  | | | | |  | | | |  | | |  | | | | |  |  |  |  |  |  |  |  |  |  |  |  |  |  |  |
|  |  |  |  |  |  |  |  |  |  |  |  |  |  |  |  |  |  |  |  |  |  |  |  |  |  |  |  |  |  |  |  |  |  |  |  |  | |  | | | | |  | | | | |  | | | |  | | |  | | | | |  |  |  |  |  |  |  |  |  |  |  |  |  |  |  |
| **22. Os moradores costumam comer algum tipo de verdura ou legume?**  ( 1 ) Sim ( 2 ) Não | | | | | | | | | | | | | | | | | | | | | | | | | | | | | | | | | | | |  | |  | | | | |  | | | | |  | | | |  | | |  | | | | |  |  |  |  |  |  |  |  |  |  |  |  |  |  |  |
|  |  |  |  |  |  |  |  |  |  |  |  |  |  |  |  |  |  |  |  |  |  |  |  |  |  |  |  |  |  |  |  |  |  |  |  |  | |  | | | | |  | | | | |  | | | |  | | |  | | | | |  |  |  |  |  |  |  |  |  |  |  |  |  |  |  |
| **23. Com qual frequência costumam comer?**   \|  \| 1 a 2 dias  Por semana \| 3 a 4 dias  Por semana \| 5 a 6 dias  Por semana \| Todos os dias  (Inclusive sábado e domingo) \| Quase nunca \| Nunca \| \| --- \| --- \| --- \| --- \| --- \| --- \| --- \| \| Macaxeira / aipim \|  \|  \|  \|  \|  \|  \| \| Batata \|  \|  \|  \|  \|  \|  \| \| Cará \|  \|  \|  \|  \|  \|  \| \| Beterraba \|  \|  \|  \|  \|  \|  \| \| Cenoura \|  \|  \|  \|  \|  \|  \| \| Abóbora / Gerimum \|  \|  \|  \|  \|  \|  \| \| Inhame \|  \|  \|  \|  \|  \|  \| \| Tomate \|  \|  \|  \|  \|  \|  \| \| Alface \|  \|  \|  \|  \|  \|  \| | | | | | | | | | | | | | | | | | | | |  | | |  | | | |  | | | | |  | | | | | | | | | | | | | | |  | | |  | | |  | | | |  | | | |  | | | | |  | |  | | |  |  |  |  |
| **24. Vocês costumam comer salada de alface e tomate ou salada de qualquer outra verdura ou legume cru?**  **Em quantos dias da semana?** | | | | | | | | | | | | | | | | | | | | | | | | | | | | | | | | | | | |  | |  | | | | |  | | | | |  | | | |  | | |  | | | | |  |  |  |  |  |  |  |  |  |  |  |  |  |  |  |
| ( 1 ) 1 a 2 dias por semana | | | | | | | | | | | | | | | | | | | |  | | |  | | | |  | | | | |  | | | | | | | | | | | | | | |  | | |  | | |  | | | |  | | | |  | | | | |  | |  | | |  |  |  |  |
| ( 2 ) 3 a 4 dias por semana | | | | | | | | | | | | | | | | | | | |  | | |  | | | |  | | | | |  | | | | | | | | | | | | | | |  | | |  | | |  | | | |  | | | |  | | | | |  | |  | | |  |  |  |  |
| ( 3 ) 5 a 6 dias por semana | | | | | | | | | | | | | | | | | | | |  | | |  | | | |  | | | | |  | | | | | | | | | | | | | | |  | | |  | | |  | | | |  | | | |  | | | | |  | |  | | |  |  |  |  |
| ( 4 ) Todos os dias (Inclusive sábado e domingo) | | | | | | | | | | | | | | | | | | | |  | | |  | | | |  | | | | |  | | | | | | | | | | | | | | |  | | |  | | |  | | | |  | | | |  | | | | |  | |  | | |  |  |  |  |
| ( 5 ) Quase nunca | | | | | | | | | | | | | | | | | | | |  | | |  | | | |  | | | | |  | | | | | | | | | | | | | | |  | | |  | | |  | | | |  | | | |  | | | | |  | |  | | |  |  |  |  |
| ( 6 ) Nunca | | | | | | | | | | | | | | | | | | | |  | | |  | | | |  | | | | |  | | | | | | | | | | | | | | |  | | |  | | |  | | | |  | | | |  | | | | |  | |  | | |  |  |  |  |
| **25. Em quantos dias da semana vocês costumam comer carne?** | | | | | | | | | | | | | | | | | | | | | | | | | | | | | | | | | | | |  | |  | | | | |  | | | | |  | | | |  | | |  | | | | |  |  |  |  |  |  |  |  |  |  |  |  |  |  |  |
| \|  \| 1 a 2 dias  Por semana \| 3 a 4 dias  Por semana \| 5 a 6 dias  Por semana \| Todos os dias  (Inclusive sábado e domingo) \| Quase nunca \| Nunca \| \| --- \| --- \| --- \| --- \| --- \| --- \| --- \| \| Boi \|  \|  \|  \|  \|  \|  \| \| Porco \|  \|  \|  \|  \|  \|  \| \| Carne de caça \|  \|  \|  \|  \|  \|  \| \| Frango \|  \|  \|  \|  \|  \|  \| \| Galinha \|  \|  \|  \|  \|  \|  \| | | | | | | | | | | | | | | | | | | | |  | | |  | | | |  | | | | |  | | | | | | | | | | | | | | |  | | |  | | |  | | | |  | | | |  | | | | |  | |  | | |  |  |  |  |
| **26. Quando vocês comem a carne vermelha com gordura, vocês costumam:** | | | | | | | | | | | | | | | | | | | | | | | | | | | | | | | | |  | |  | | | | |  | | | | |  | | | |  | | |  |  |  |  |  |  |  |  |  |  |  |  |  |  |  |  |  |  |  |  |  |  |  |
| ( 1 ) Tirar sempre o excesso de gordura ( 2 ) Comer com a gordura ( 3 ) Não come carne vermelha com muita gordura | | | | | | | | | | | | | | | | | | | |  | | |  | | | |  | | | | |  | | | | | | | | | | | | | | |  | | |  | | |  | | | |  | | | |  | | | | |  | |  | | |  |  |  |  |
|  |  |  |  |  |  |  |  |  |  |  |  |  |  |  |  |  |  |  |  |  | | |  | | | |  | | | | |  | | | | | | | | | | | | | | |  | | |  | | |  | | | |  | | | |  | | | | |  | |  | | |  |  |  |  |
|  |  |  |  |  |  |  |  |  |  |  |  |  |  |  |  |  |  |  |  |  | | |  | | | |  | | | | |  | | | | | | | | | | | | | | |  | | |  | | |  | | | |  | | | |  | | | | |  | |  | | |  |  |  |  |
| **27. Quando vocês comem o frango/galinha com pele, vocês costumam:** | | | | | | | | | | | | | | | | | | | | | | | | | | | | | | | | |  | |  | | | | |  | | | | |  | | | |  | | |  |  |  |  |  |  |  |  |  |  |  |  |  |  |  |  |  |  |  |  |  |  |  |
| ( 1 ) Tirar sempre a pele | | | | | | | | | | | | | | | | | | | | | |  | | | |  | | | | |  | | | | | | | |  | | | | |  | | | | | | | | | | | | | | |  | | | |  | | | |  | | |  | |  |  |  |
| ( 2 ) Comer com a pele | | | | | | | | | | | | | | | | | | | | | |  | | | |  | | | | |  | | | | | | | |  | | | | |  | | | | | | | | | | | | | | |  | | | |  | | | |  | | |  | |  |  |  |
| ( 3 ) Não come pedaços de frango com pele | | | | | | | | | | | | | | | | | | | |  | | |  | | | |  | | | | |  | | | | | | | | | | | | | | |  | | |  | | |  | | | |  | | | |  | | | | |  | |  | | |  |  |  |  |
|  | |  | | |  | | | | |  | | | | | | | | | | | |  | | | |  | | | | |  | | | | | | | |  | | | | |  | | | | | | | | | | | | | | |  | | | |  | | | |  | | |  | |  |  |  |
| **28. Em quantos dias da semana vocês costumam comer peixe?** | | | | | | | | | | | | | | | | | | | | | | | | | | | | | | | | |  | |  | | | | |  | | | | |  | | | |  | | |  |  |  |  |  |  |  |  |  |  |  |  |  |  |  |  |  |  |  |  |  |  |  |
| ( 1 ) 1 a 2 dias por semana | | | | | | | | | | | | | | | | | | | |  | | |  | | | |  | | | | |  | | | | | | | | | | | | | | |  | | |  | | |  | | | |  | | | |  | | | | |  | |  | | |  |  |  |  |
| ( 2 ) 3 a 4 dias por semana | | | | | | | | | | | | | | | | | | | |  | | |  | | | |  | | | | |  | | | | | | | | | | | | | | |  | | |  | | |  | | | |  | | | |  | | | | |  | |  | | |  |  |  |  |
| ( 3 ) 5 a 6 dias por semana | | | | | | | | | | | | | | | | | | | |  | | |  | | | |  | | | | |  | | | | | | | | | | | | | | |  | | |  | | |  | | | |  | | | |  | | | | |  | |  | | |  |  |  |  |
| ( 4 ) Todos os dias (Inclusive sábado e domingo) | | | | | | | | | | | | | | | | | | | |  | | |  | | | |  | | | | |  | | | | | | | | | | | | | | |  | | |  | | |  | | | |  | | | |  | | | | |  | |  | | |  |  |  |  |
| ( 5 ) Quase nunca | | | | | | | | | | | | | | | | | | | |  | | |  | | | |  | | | | |  | | | | | | | | | | | | | | |  | | |  | | |  | | | |  | | | |  | | | | |  | |  | | |  |  |  |  |
| ( 6 ) Nunca | | | | | | | | | | | | | | | | | | | |  | | |  | | | |  | | | | |  | | | | | | | | | | | | | | |  | | |  | | |  | | | |  | | | |  | | | | |  | |  | | |  |  |  |  |
|  | |  | | |  | | | | |  | | | | | | | | | | | |  | | | |  | | | | |  | | | | | | | |  | | | | |  | | | | | | | | | | | | | | |  | | | |  | | | |  | | |  | |  |  |  |
| **29. Quando vocês comem o peixe, vocês costumam:** | | | | | | | | | | | | | | | | | | | | | | | | | | | | | | | | |  | |  | | | | |  | | | | |  | | | |  | | |  |  |  |  |  |  |  |  |  |  |  |  |  |  |  |  |  |  |  |  |  |  |  |
| ( 1 ) Comer frito | | | | | | | | | | | | | | | | | | | | | |  | | | |  | | | | |  | | | | | | | |  | | | | |  | | | | | | | | | | | | | | |  | | | |  | | | |  | | |  | |  |  |  |
| ( 2 ) Comer cozido | | | | | | | | | | | | | | | | | | | | | |  | | | |  | | | | |  | | | | | | | |  | | | | |  | | | | | | | | | | | | | | |  | | | |  | | | |  | | |  | |  |  |  |
| ( 3 ) Comer assado | | | | | | | | | | | | | | | | | | | |  | | |  | | | |  | | | | |  | | | | | | | | | | | | | | |  | | |  | | |  | | | |  | | | |  | | | | |  | |  | | |  |  |  |  |
|  | |  | | |  | | | | |  | | | | | | | | | | | |  | | | |  | | | | |  | | | | | | | |  | | | | |  | | | | | | | | | | | | | | |  | | | |  | | | |  | | |  | |  |  |  |
| **30. Em quantos dias da semana vocês costumam consumir?** | | | | | | | | | | | | | | | | | | | | | | | | | | | | | | | | |  | |  | | | | |  | | | | |  | | | |  | | |  |  |  |  |  |  |  |  |  |  |  |  |  |  |  |  |  |  |  |  |  |  |  |
| \|  \| 1 a 2 dias  Por semana \| 3 a 4 dias  Por semana \| 5 a 6 dias  Por semana \| Todos os dias  (Inclusive sábado e domingo) \| Quase nunca \| Nunca \| \| --- \| --- \| --- \| --- \| --- \| --- \| --- \| \| Frutas \|  \|  \|  \|  \|  \|  \| \| Suco de frutas \|  \|  \|  \|  \|  \|  \| \| Suco Artificial (caixa ou saco) \|  \|  \|  \|  \|  \|  \| \| Refrigerante \|  \|  \|  \|  \|  \|  \| | | | | | | | | | | | | | | | | | | | |  | | |  | | | |  | | | | |  | | | | | | | | | | | | | | |  | | |  | | |  | | | |  | | | |  | | | | |  | |  | | |  |  |  |  |
| **31. Especifique o tipo:** | | | | |  | | | | |  | | | | | | | | | | | |  | | | |  | | | | |  | | | | | | | |  | | | | |  | | | | | | | | | | | | | | |  | | | |  | | | |  | | |  | |  |  |  |
| ( 1 ) Normal | | | | |  | | | | |  | | | | | | | | | | | |  | | | |  | | | | |  | | | | | | | |  | | | | |  | | | | | | | | | | | | | | |  | | | |  | | | |  | | |  | |  |  |  |
| ( 2 ) Diet/Light/Zero | | | | |  | | | | |  | | | | | | | | | | | |  | | | |  | | | | |  | | | | | | | |  | | | | |  | | | | | | | | | | | | | | |  | | | |  | | | |  | | |  | |  |  |  |
| ( 3 ) Ambos | | | | |  | | | | |  | | | | | | | | | | | |  | | | |  | | | | |  | | | | | | | |  | | | | |  | | | | | | | | | | | | | | |  | | | |  | | | |  | | |  | |  |  |  |
| ( 4 ) Não sei | | | | |  | | | | |  | | | | | | | | | | | |  | | | |  | | | | |  | | | | | | | |  | | | | |  | | | | | | | | | | | | | | |  | | | |  | | | |  | | |  | |  |  |  |
|  | |  | | |  | | | | |  | | | | | | | | | | | |  | | | |  | | | | |  | | | | | | | |  | | | | |  | | | | | | | | | | | | | | |  | | | |  | | | |  | | |  | |  |  |  |
| **32. Em quantos dias da semana vocês costumam comer alimentos doces, tais como:**   \|  \| 1 a 2 dias  Por semana \| 3 a 4 dias  Por semana \| 5 a 6 dias  Por semana \| Todos os dias  (Inclusive sábado e domingo) \| Quase nunca \| Nunca \| \| --- \| --- \| --- \| --- \| --- \| --- \| --- \| \| Sorvetes \|  \|  \|  \|  \|  \|  \| \| Chocolates \|  \|  \|  \|  \|  \|  \| \| Bolos \|  \|  \|  \|  \|  \|  \| \| Biscoitos \|  \|  \|  \|  \|  \|  \| \| Doces \|  \|  \|  \|  \|  \|  \| | | | | | | | | | | | | | | | | | | | | | | | | | | | | | | | | |  | |  | | | | |  | | | | |  | | | |  | | |  |  |  |  |  |  |  |  |  |  |  |  |  |  |  |  |  |  |  |  |  |  |  |
| **33. Em quantos dias da semana vocês costumam trocar a comida do almoço ou jantar por sanduíches,**  **salgados e/ou pizza?** | | | | | | | | | | | | | | | | | | | | | | | | | | | | | | | | |  | |  | | | | |  | | | | |  | | | |  | | |  |  |  |  |  |  |  |  |  |  |  |  |  |  |  |  |  |  |  |  |  |  |  |
| ( 1 ) 1 a 2 dias por semana | | | | | | | | | | | | | | | | | | | |  | | |  | | | |  | | | | |  | | | | | | | | | | | | | | |  | | |  | | |  | | | |  | | | |  | | | | |  | |  | | |  |  |  |  |
| ( 2 ) 3 a 4 dias por semana | | | | | | | | | | | | | | | | | | | |  | | |  | | | |  | | | | |  | | | | | | | | | | | | | | |  | | |  | | |  | | | |  | | | |  | | | | |  | |  | | |  |  |  |  |
| ( 3 ) 5 a 6 dias por semana | | | | | | | | | | | | | | | | | | | |  | | |  | | | |  | | | | |  | | | | | | | | | | | | | | |  | | |  | | |  | | | |  | | | |  | | | | |  | |  | | |  |  |  |  |
| ( 4 ) Todos os dias (Inclusive sábado e domingo) | | | | | | | | | | | | | | | | | | | |  | | |  | | | |  | | | | |  | | | | | | | | | | | | | | |  | | |  | | |  | | | |  | | | |  | | | | |  | |  | | |  |  |  |  |
| ( 5 ) Quase nunca | | | | | | | | | | | | | | | | | | | |  | | |  | | | |  | | | | |  | | | | | | | | | | | | | | |  | | |  | | |  | | | |  | | | |  | | | | |  | |  | | |  |  |  |  |
| ( 6 ) Nunca | | | | | | | | | | | | | | | | | | | |  | | |  | | | |  | | | | |  | | | | | | | | | | | | | | |  | | |  | | |  | | | |  | | | |  | | | | |  | |  | | |  |  |  |  |
| **34. Em quantos dias da semana vocês costumam comer produtos industrializados?** | | | | | | | | | | | | | | | | | | | | | | | | | | | | | | | | |  | |  | | | | |  | | | | |  | | | |  | | |  |  |  |  |  |  |  |  |  |  |  |  |  |  |  |  |  |  |  |  |  |  |  |
| \|  \| 1 a 2 dias  Por semana \| 3 a 4 dias  Por semana \| 5 a 6 dias  Por semana \| Todos os dias  (Inclusive sábado e domingo) \| Quase nunca \| Nunca \| \| --- \| --- \| --- \| --- \| --- \| --- \| --- \| \| Sardinha \|  \|  \|  \|  \|  \|  \| \| Salame \|  \|  \|  \|  \|  \|  \| \| Salsicha \|  \|  \|  \|  \|  \|  \| \| Almôdegas \|  \|  \|  \|  \|  \|  \| \| Feijoada \|  \|  \|  \|  \|  \|  \| \| Bacon \|  \|  \|  \|  \|  \|  \| \| Calabreza \|  \|  \|  \|  \|  \|  \| | | | | | | | | | | | | | | | | | | | |  | | |  | | | |  | | | | |  | | | | | | | | | | | | | | |  | | |  | | |  | | | |  | | | |  | | | | |  | |  | | |  |  |  |  |
| **Avaliação de Antecedentes para a Hipertensão Arterial, Doenças Cardiovasculares e Condições de Saúde** | | | | | | | | | | | | | | | | | | | | | | | | | | | | |  |  |  |  |  |  |  |  |  |  |  |  |  |  |  |  |  |  |  |  |  |  |  |  |  |  |  |  |  |  |  |  |  |  |  |  |  |  |  |  |  |  |  |  |  |  |
|  |  | |  |  | |  | | |  | |  |  | | |  | | |  | | | | | | | | | |  | | | | | |  | | | | | | | |  | | | | | | | | | | | | | |  | | | | | | | |  | | | | |  | | | | | |
| **35. O Sr (a) tem ou já teve problema de pressão alta?** (SE A RESPOSTA FOR 2, NÃO MARCAR ITEM 51, 55, 56) | | | | | | | | |  | | | | | | | | | | | | | | | | | | | | | | | | | | | | | | | | | | | | | | | | | | | | | | | | | | | | | | | | | | | |  | | | | | |
| ( 1 ) Sim ( 2 ) Não ( 3 ) Não sabe | | | | | | | | |  | |  |  | | |  | | |  | | | | | | | | | |  | | | | | |  | | | | | | | |  | | | | | | | | | | | | | |  | | | | | | | |  | | | | |  | | | | | |
| **36. Algum profissional da área de saúde já te disse que você problema de pressão alta?** | | | | | | | | | | | | | | | | | | | | | | | | | | | | | | | | | | | | | | | | | | | | | | | | | | | | | | | | | | | | | | | | | | | | | | | | | | |
| ( 1 ) Sim ( 2 ) Não ( 3 ) Não sabe | | | | | | | | | | | | | | | | | | | | | | | | | | | | | | | | | | | | | | | | | | | | | | | | | | | | | | | | | | | | | | | | | | | | | | | | | | |
| **37. Se sim, quem?**  ( 1 ) Médico ( 2 ) Enfermeiro ( 3 ) Farmacêutico  ( 4 ) Outro, especificar:___________________________________ | | | | | | | | | | | | | | | | | | | | | | | | | | | | | | | | | | | | | | | | | | | | | | | | | | | | | | | | | | | | | | | | | | | | | | | | | | |
| **38. Está fazendo acompanhamento em serviço de saúde para a pressão alta?** | | | | | | | | | | | | | | | | | | | | | | | | | | | | | | | | | | | | | | | | | | | | | | | | | | | | | | | | | | | | | | | | | | | | | | | | | | |
| ( 1 ) Sim ( 2 ) Não | | | | | | | | |  | |  |  | | |  | | |  | | | | | | | | | |  | | | | | |  | | | | | | | |  | | | | | | | | | | | | | |  | | | | | | | |  | | | | |  | | | | | |
| **38. Se sim, onde?**_______________________________________ | | | | | | | | | | | | | | | | | | | | | | | | | | | |  | | | | | |  | | | | | | | |  | | | | | | | | | | | | | |  | | | | | | | |  | | | | |  | | | | | |
| **39. O Sr (a) fuma ou fumou?** | | | | | | | | | | | | | | | | | | | | | | | | | | | |  | | | | | |  | | | | | | | |  | | | | | | | | | | | | | |  | | | | | | | |  | | | | |  | | | | | |
| ( 1 ) Sim ( 2 ) Não ( 3 ) Parou, há ___________(tempo) | | | | | | | | | | | |  | | |  | | |  | | | | | | | | | |  | | | | | |  | | | | | | | |  | | | | | | | | | | | | | |  | | | | | | | |  | | | | |  | | | | | |
| **40. Se sim, por quanto tempo?** Anos_______e/ou meses________ | | | | | | | | | | | | | | | | | | | | | | | | | | | |  | | | | | |  | | | | | | | |  | | | | | | | | | | | | | |  | | | | | | | |  | | | | |  | | | | | |
| **41. Quantos maço fuma por dia?** | | | | | |  | | |  | |  |  | | |  | | |  | | | | | | | | | |  | | | | | |  | | | | | | | |  | | | | | | | | | | | | | |  | | | | | | | |  | | | | |  | | | | | |
| ( 1 ) Menos que um ( 2 ) Um ( 3 ) Dois ( 4 ) Mais que dois ( 5 ) Não sabe informar | | | | | | | | | | | | | | | | | | | | | | | | | | | |  | | | | | |  | | | | | | | |  | | | | | | | | | | | | | |  | | | | | | | |  | | | | |  | | | | | |
|  |  | |  |  | |  | | |  | |  |  | | |  | | |  | | | | | | | | | |  | | | | | |  | | | | | | | |  | | | | | | | | | | | | | |  | | | | | | | |  | | | | |  | | | | | |
| **42. O Sr (a) consome ou consumia bebidas alcoólicas?** | | | | | | | | | | | | | | | | | | | | | | | | | | | |  | | | | | |  | | | | | | | |  | | | | | | | | | | | | | |  | | | | | | | |  | | | | |  | | | | | |
| ( 1 ) Sim especificar_______anos e/ou meses _______ ( 2 ) Não | | | | | | | | | | | | | | | | | | | | | | | | | | | |  | | | | | |  | | | | | | | |  | | | | | | | | | | | | | |  | | | | | | | |  | | | | |  | | | | | |
|  |  | |  |  | |  | | |  | |  |  | | |  | | |  | | | | | | | | | |  | | | | | |  | | | | | | | |  | | | | | | | | | | | | | |  | | | | | | | |  | | | | |  | | | | | |
| **43. A Sra usa ou já fez uso de pílula ou hormônio anticoncepcional?** (SOMENTE PARA MULHERES ) | | | | | | | | | | | | | | | | | | | | | | | | | | | |  | | | | | |  | | | | | | | |  | | | | | | | | | | | | | |  | | | | | | | |  | | | | |  | | | | | |
| ( 1 ) Sim ( 2 ) Não ( 3 ) Parou, há ___________(tempo) | | | | | | | | | | | | | | | | | | | | | | | | | | | |  | | | | | |  | | | | | | | |  | | | | | | | | | | | | | |  | | | | | | | |  | | | | |  | | | | | |
|  |  | |  |  | |  | | |  | |  |  | | |  | | |  | | | | | | | | | |  | | | | | |  | | | | | | | |  | | | | | | | | | | | | | |  | | | | | | | |  | | | | |  | | | | | |
| **44. O Sr (a) tem algum parente com:** | | | | | | | | | | | | | | | | | | | | | | | | | | | |  | | | | | |  | | | | | | | |  | | | | | | | | | | | | | |  | | | | | | | |  | | | | |  | | | | | |
|  |  | | Sim | Não | | Não Sabe | | |  | |  |  | | |  | | |  | | | | | | | | | |  | | | | | |  | | | | | | | |  | | | | | | | | | | | | | |  | | | | | | | |  | | | | |  | | | | | |
| 44.1 Pressão Alta | | | 1 | 2 | | 3 | | |  | |  |  | | |  | | |  | | | | | | | | | |  | | | | | |  | | | | | | | |  | | | | | | | | | | | | | |  | | | | | | | |  | | | | |  | | | | | |
| 44.2 Problema de Coração | | | 1 | 2 | | 3 | | |  | |  |  | | |  | | |  | | | | | | | | | |  | | | | | |  | | | | | | | |  | | | | | | | | | | | | | |  | | | | | | | |  | | | | |  | | | | | |
| 44.3 Derrame/ AVC | | | 1 | 2 | | 3 | | |  | |  |  | | |  | | |  | | | | | | | | | |  | | | | | |  | | | | | | | |  | | | | | | | | | | | | | |  | | | | | | | |  | | | | |  | | | | | |
| 44.4 Diabetes | | | 1 | 2 | | 3 | | |  | |  |  | | |  | | |  | | | | | | | | | |  | | | | | |  | | | | | | | |  | | | | | | | | | | | | | |  | | | | | | | |  | | | | |  | | | | | |
| 44.5 Aumento Colesterol | | | 1 | 2 | | 3 | | |  | |  |  | | |  | | |  | | | | | | | | | |  | | | | | |  | | | | | | | |  | | | | | | | | | | | | | |  | | | | | | | |  | | | | |  | | | | | |
| **44.6 Se sim, quem?** (PODE MARCAR MAIS DE UMA ALTERNATIVA) | | | | | | | | | | | | | | | | | | | | | | | | | | | |  | | | | | |  | | | | | | | |  | | | | | | | | | | | | | |  | | | | | | | |  | | | | |  | | | | | |
| ( 1 ) Pai ( 2 ) Mãe ( 3 ) Avós ( 4 ) Tios ( 5 ) Irmãos ( 6 ) Filhos | | | | | | | | | | | | | | | | | | | | | | | | | | | |  | | | | | |  | | | | | | | |  | | | | | | | | | | | | | |  | | | | | | | |  | | | | |  | | | | | |
|  |  | |  |  | |  | | |  | |  |  | | |  | | |  | | | | | | | | | |  | | | | | |  | | | | | | | |  | | | | | | | | | | | | | |  | | | | | | | |  | | | | |  | | | | | |
| **45. O Sr (a). Tem ou já teve problema de coração?** | | | | | | | | | | | | | | | | | | | | | | | | | | | |  | | | | | |  | | | | | | | |  | | | | | | | | | | | | | |  | | | | | | | |  | | | | |  | | | | | |
| ( 1 ) Sim ( 2 ) Não ( 3 ) Não sabe | | | | | | | | | | | | | | | | | | | | | | | | | | | |  | | | | | |  | | | | | | | |  | | | | | | | | | | | | | |  | | | | | | | |  | | | | |  | | | | | |
| **46. O Sr (a). Tem ou já teve derrame/AVC?** | | | | | | | | | | | | | | | | | | | | | | | | | | | |  | | | | | |  | | | | | | | |  | | | | | | | | | | | | | |  | | | | | | | |  | | | | |  | | | | | |
| ( 1 ) Sim ( 2 ) Não ( 3 ) Não sabe | | | | | | | | | | | | | | | | | | | | | | | | | | | |  | | | | | |  | | | | | | | |  | | | | | | | | | | | | | |  | | | | | | | |  | | | | |  | | | | | |
|  |  | |  |  | |  | | |  | |  |  | | |  | | |  | | | | | | | | | |  | | | | | |  | | | | | | | |  | | | | | | | | | | | | | |  | | | | | | | |  | | | | |  | | | | | |
| **47. O Sr (a). Tem ou já teve Diabetes?** ( 1 ) Sim ( 2 ) Não ( 3 ) Não sabe | | | | | | | | | | | | | | | | | | | | | | | | | | | |  | | | | | |  | | | | | | | |  | | | | | | | | | | | | | |  | | | | | | | |  | | | | |  | | | | | |
|  | | | | | | | | | | | | | | | | | | | | | | | | | | | |  | | | | | |  | | | | | | | |  | | | | | | | | | | | | | |  | | | | | | | |  | | | | |  | | | | | |
| **47.1 Se sim, toma medicamentos?** ( 1 ) Sim ( 2 ) Não (ANOTAR SE A RESPOSTA DO ITEM 47 FOR SIM) | | | | | | | | | | | | | | | | | | | | | | | | | | | |  | | | | | |  | | | | | | | |  | | | | | | | | | | | | | |  | | | | | | | |  | | | | |  | | | | | |
|  |  | |  |  | |  | | |  | |  |  | | |  | | |  | | | | | | | | | |  | | | | | |  | | | | | | | |  | | | | | | | | | | | | | |  | | | | | | | |  | | | | |  | | | | | |
| **47.1.1 Se toma medicamentos,** (ANOTAR SE A RESPOSTA DO ITEM 47.1 FOR SIM) | | | | | | | | | | | | | | | | | | | | | | | | | | | |  | | | | | |  | | | | | | | |  | | | | | | | | | | | | | |  | | | | | | | |  | | | | |  | | | | | |
| ( 1 ) Especifique o nome: __________________________________ ( 2 ) Não sabe | | | | | | | | | | | | | | | | | | | | | | | | | | | | | | | | | | | | | | | | | | | | | | | | | | | | | | | | | | | | | | | | | | | | |  | | | | | |
|  | | | | | | | | | | | | | | | | | | | | | | | | | | | |  | | | | | |  | | | | | | | |  | | | | | | | | | | | | | |  | | | | | | | |  | | | | |  | | | | | |
| **47.2 O Sr (a). já mediu a glicemia alguma vez?** | | | | | | | | | | | | | | | | | | | | | | | | | | | |  | | | | | |  | | | | | | | |  | | | | | | | | | | | | | |  | | | | | | | |  | | | | |  | | | | | |
| ( 1 ) Sim ( 2 ) Não ( 3 ) Não sabe | | | | | | | | | | | | | | | | | | | | | | | | | | | |  | | | | | |  | | | | | | | |  | | | | | | | | | | | | | |  | | | | | | | |  | | | | |  | | | | | |
|  |  | |  |  | |  | | |  | |  |  | | |  | | |  | | | | | | | | | |  | | | | | |  | | | | | | | |  | | | | | | | | | | | | | |  | | | | | | | |  | | | | |  | | | | | |
| **47.2.1 Se sim, lembra quando foi a última medida?**  (ANOTAR SE A RESPOSTA DO ITEM 47.2 FOR SIM) | | | | | | | | | | | | | | | | | | | | | | | | | | | |  | | | | | |  | | | | | | | |  | | | | | | | | | | | | | |  | | | | | | | |  | | | | |  | | | | | |
| ( 1 ) Últimos 6 meses ( 2 ) Último 12 meses ( 3 ) Mais 1 ano | | | | | | | | | | | | | | | | | | | | | | | | | | | |  | | | | | |  | | | | | | | |  | | | | | | | | | | | | | |  | | | | | | | |  | | | | |  | | | | | |
| **48. O Sr (a). Tem ou já teve Dislipidemias (Colesterol elevado, Triglicérides)?** | | | | | | | | | | | | | | | | | | | | | | | | | | | |  | | | | | |  | | | | | | | |  | | | | | | | | | | | | | |  | | | | | | | |  | | | | |  | | | | | |
| ( 1 ) Sim ( 2 ) Não ( 3 ) Não sabe | | | | | | | | | | | | | | | | | | | | | | | | | | | |  | | | | | |  | | | | | | | |  | | | | | | | | | | | | | |  | | | | | | | |  | | | | |  | | | | | |
|  |  | |  |  | |  | | |  | |  |  | | |  | | |  | | | | | | | | | |  | | | | | |  | | | | | | | |  | | | | | | | | | | | | | |  | | | | | | | |  | | | | |  | | | | | |
| **48.1 Se sim, toma medicamentos?** ( 1 ) Sim ( 2 ) Não ( 3 ) Não sabe (ANOTAR SE A RESPOSTA DO ITEM 48 FOR SIM) | | | | | | | | | | | | | | | | | | | | | | | | | | | |  | | | | | |  | | | | | | | |  | | | | | | | | | | | | | |  | | | | | | | |  | | | | |  | | | | | |
|  |  | |  |  | |  | | |  | |  |  | | |  | | |  | | | | | | | | | |  | | | | | |  | | | | | | | |  | | | | | | | | | | | | | |  | | | | | | | |  | | | | |  | | | | | |
| **48.1.1 Se toma medicamentos,** (ANOTAR SE A RESPOSTA DO ITEM 48.1 FOR SIM) | | | | | | | | | | | | | | | | | | | | | | | | | | | |  | | | | | |  | | | | | | | |  | | | | | | | | | | | | | |  | | | | | | | |  | | | | |  | | | | | |
| ( 1 ) Especifique o nome: ___________________________________ ( 2 ) Não sabe | | | | | | | | | | | | | | | | | | | | | | | | | | | | | | | | | | | | | | | | | | | | | | | | | | | | | | | | | | | | | | | | | | | | | | | | | | |
|  |  | |  |  | |  | | |  | |  |  | | |  | | |  | | | | | | | | | |  | | | | | |  | | | | | | | |  | | | | | | | | | | | | | |  | | | | | | | |  | | | | |  | | | | | |
| **48.2 O Sr (a). já mediu o colesterol, triglicérides no sangue, alguma vez?** | | | | | | | | | | | | | | | | | | | | | | | | | | | |  | | | | | |  | | | | | | | |  | | | | | | | | | | | | | |  | | | | | | | |  | | | | |  | | | | | |
| ( 1 ) Sim ( 2 ) Não ( 3 ) Não sabe | | | | | | | | | | | | | | | | | | | | | | | | | | | |  | | | | | |  | | | | | | | |  | | | | | | | | | | | | | |  | | | | | | | |  | | | | |  | | | | | |
| **48.2.1 Se sim, lembra quando foi a última medida?**  (ANOTAR SE A RESPOSTA DO ITEM 48.2 FOR SIM) | | | | | | | | | | | | | | | | | | | | | | | | | | | |  | | | | | |  | | | | | | | |  | | | | | | | | | | | | | |  | | | | | | | |  | | | | |  | | | | | |
| ( 1 ) Últimos 6 meses ( 2 ) Último 12 meses ( 3 ) Mais 1 ano | | | | | | | | | | | | | | | | | | | | | | | | | | | |  | | | | | |  | | | | | | | |  | | | | | | | | | | | | | |  | | | | | | | |  | | | | |  | | | | | |
|  |  | |  |  | |  | | |  | |  |  | | |  | | |  | | | | | | | | | |  | | | | | |  | | | | | | | |  | | | | | | | | | | | | | |  | | | | | | | |  | | | | |  | | | | | |
| **49. O Sr (a) tem hábito de medir a pressão?** ( 1 ) Sim ( 2 ) Não | | | | | | | | | | | | | | | | | | | | | | | | | | | |  | | | | | |  | | | | | | | |  | | | | | | | | | | | | | |  | | | | | | | |  | | | | |  | | | | | |
|  |  | |  |  | |  | | |  | |  |  | | |  | | |  | | | | | | | | | |  | | | | | |  | | | | | | | |  | | | | | | | | | | | | | |  | | | | | | | |  | | | | |  | | | | | |
| **49.1 Se sim, qual a frequência?**  ( 1 ) Diariamente ( 2 ) Semanalmente | | | | | | | | | | | | | | | | | | | | | | | | | | | |  | | | | | |  | | | | | | | |  | | | | | | | | | | | | | |  | | | | | | | |  | | | | |  | | | | | |
| ( 3 ) Mensalmente ( 4 ) Semestralmente ( 5 ) Anualmente  ( 6 ) Outro especificar_____________________________________ | | | | | | | | | | | | | | | | | | | | | | | | | | | |  | | | | | |  | | | | | | | |  | | | | | | | | | | | | | |  | | | | | | | |  | | | | |  | | | | | |
| **49.2 Se sim, onde costuma medir a sua pressão?**  (PODE MARCAR MAIS DE UMA ALTERNATIVA) | | | | | | | | | | | | | | | | | | | | | | | | | | | |  | | | | | |  | | | | | | | |  | | | | | | | | | | | | | |  | | | | | | | |  | | | | |  | | | | | |
| ( 1 ) Pólo Base ( 2 ) UBS ( 3 ) Farmácia ( 4 ) Hospital | | | | | | | | | | | | | | | | | | | | | | | | | | | |  | | | | | |  | | | | | | | |  | | | | | | | | | | | | | |  | | | | | | | |  | | | | |  | | | | | |
| ( 5 ) Médico particular ( 6 ) Médico convênio ( 7 ) Em casa  ( 8 ) Outro, especificar:____________________________________ | | | | | | | | | | | | | | | | | | | | | | | | | | | |  | | | | | |  | | | | | | | |  | | | | | | | | | | | | | |  | | | | | | | |  | | | | |  | | | | | |
| **50. Você sabe quando foi a última vez que você mediu a pressão?** | | | | | | | | | | | | | | | | | | | | | | | | | | | |  | | | | | |  | | | | | | | |  | | | | | | | | | | | | | |  | | | | | | | |  | | | | |  | | | | | |
| ( 1 ) Sim, especificar em meses:______e/ou anos ______e/ou dias ( 2 ) Não | | | | | | | | | | | | | | | | | | | | | | | | | | | |  | | | | | |  | | | | | | | |  | | | | | | | | | | | | | |  | | | | | | | |  | | | | |  | | | | | |
|  | | | | | | | | | | | | | | | | | | | | | | | | | | | |  | | | | | |  | | | | | | | |  | | | | | | | | | | | | | |  | | | | | | | |  | | | | |  | | | | | |
| **50.1 Você sabe qual o valor da última medida de pressão?**  (ANOTAR COMO RELATADO) | | | | | | | | | | | | | | | | | | | | | | | | | | | |  | | | | | |  | | | | | | | |  | | | | | | | | | | | | | |  | | | | | | | |  | | | | |  | | | | | |
| ( 1 ) Sim, Sistólica:_________mmHg X Diatólica:________mmHg ( 2 ) Não | | | | | | | | | | | | | | | | | | | | | | | | | | | |  | | | | | | | | | | | | | |  | | | | | | | | | | | | | |  | | | | | | | | | | | | |  | | | | | |
| **51. Há quanto tempo que você tem pressão alta?** (ANOTAR SE A RESPOSTA DO ITEM 1 FOR SIM) | | | | | | | | | | | | | | | | | | | | | | | | | | | |  | | | | | | | | | | | | | |  | | | | | | | | | | | | | | | | | | | | | | | | | | |  | | | | | |
| ( 1 ) Especificar em anos:______e/ou meses ______e/ou dias______ | | | | | | | | | | | | | | | | | | | | | | | | | | | |  | | | | | |  | | | | | | | |  | | | | | | | | | | | | | |  | | | | | | | |  | | | | |  | | | | | |
| **52. Alguém lhe indicou algum tratamento com remédio?** ( 1 ) Sim ( 2 ) Não | | | | | | | | | | | | | | | | | | | | | | | | | | | | | | | | | | | | | | | | | | | | | | | | | | | | | | | |  | | | | | | | |  | | | | |  | | | | | |
| **52.1 Se sim, quem?** (PODE MARCAR MAIS DE UMA ALTERNATIVA) | | | | | | | | | | | | | | | | | | | | | | | | | | | |  | | | | | |  | | | | | | | |  | | | | | | | | | | | | | |  | | | | | | | |  | | | | |  | | | | | |
| ( 1 ) Médico ( 2 ) Enfermeiro ( 3 ) Farmacêutico  ( 4 ) Outro, especificar__________________________________ | | | | | | | | | | | | | | | | | | | | | | | | | | | |  | | | | | |  | | | | | | | |  | | | | | | | | | | | | | |  | | | | | | | |  | | | | |  | | | | | |
|  |  | |  |  | |  | | |  | |  |  | | |  | | |  | | | | | | | | | |  | | | | | |  | | | | | | | |  | | | | | | | | | | | | | |  | | | | | | | |  | | | | |  | | | | | |
| **53. Você está fazendo tratamento (medicamento ou não) para pressão alta?**( 1 ) Sim ( 2 ) Não | | | | | | | | | | | | | | | | | | | | | | | | | | | | | | | | | | | | | | | | | | | | | | | | | | | | | | | |  | | | | | | | |  | | | | |  | | | | | |
|  |  | |  |  | |  | | |  | |  |  | | |  | | |  | | | | | | | | | |  | | | | | |  | | | | | | | |  | | | | | | | | | | | | | |  | | | | | | | |  | | | | |  | | | | | |
| **53.1 Se sim, quanto tempo de tratamento?** (ANOTAR SE A RESPOSTA DO ITEM 53 FOR SIM) | | | | | | | | | | | | | | | | | | | | | | | | | | | | | | | | | | | | | | | | | | | | | | | | | | | | | | | |  | | | | | | | |  | | | | |  | | | | | |
| ( 1 ) Especificar em anos:______e/ou meses ______e/ou dias______  ( 2) Não sabe | | | | | | | | | | | | | | | | | | | | | | | | | | | | | | | | | | | | | | | | | | | | | | | | | | | | | | | |  | | | | | | | |  | | | | |  | | | | | |
|  |  | |  |  | |  | | |  | |  |  | | |  | | |  | | | | | | | | | |  | | | | | |  | | | | | | | |  | | | | | | | | | | | | | |  | | | | | | | |  | | | | |  | | | | | |
| **53.2 Que tipo de tratamento está fazendo?** | | | | | | | | | | | | | | | | | | | | | | | | | | | | | | | | | | | | | | | | | | | | | | | | | | | | | | | |  | | | | | | | |  | | | | |  | | | | | |
| ( 1 ) Com remédio ( 2 ) Sem remédio ( 3 ) Não sabe | | | | | | | | | | | | | | | | | | | | | | | | | | | | | | | | | | | | | | | | | | | | | | | | | | | | | | | |  | | | | | | | |  | | | | |  | | | | | |
|  |  | |  |  | |  | | |  | |  |  | | |  | | |  | | | | | | | | | |  | | | | | |  | | | | | | | |  | | | | | | | | | | | | | |  | | | | | | | |  | | | | |  | | | | | |
| **53.2.1 Caso esteja tomando algum remédio para pressão alta sabe o nome dos medicamentos?** | | | | | | | | | | | | | | | | | | | | | | | | | | | | | | | | | | | | | | | | | | | | | | | | | | | | | | | |  | | | | | | | |  | | | | |  | | | | | |
| ( 1 ) Sim ( 2 ) Não ( 3 ) Não se lembra todos, apenas alguns. Especifique:________________________ | | | | | | | | | | | | | | | | | | | | | | | | | | | | | | | | | | | | | | | | | | | | | | | | | | | | | | | | | | | | | | | | | | | | | | | | | | |
| **53.3 Aonde obtém os remédios?** | | | | | | | | | | | | | | | | | | | | | | | | | | | | | | | | | | | | | | | | | | | | | | | | | | | | | | | |  | | | | | | | |  | | | | |  | | | | | |
| ( 1 ) Pólo Base ( 2 ) UBS ( 3 ) Compra ( 4 ) Manda manipular  ( 5 ) Outros_________________________________ | | | | | | | | | | | | | | | | | | | | | | | | | | | | | | | | | | | | | | | | | | | | | | | | | | | | | | | |  | | | | | | | |  | | | | |  | | | | | |
| **53.5 Por onde você está fazendo tratamento para pressão alta?** | | | | | | | | | | | | | | | | | | | | | | | | | | | | | | | | | | | | | | | | | | | | | | | | | | | | | | | |  | | | | | | | |  | | | | |  | | | | | |
| ( 1 ) Pólo Base ( 2 ) UBS ( 3 ) Farmácia ( 4 ) Hospital | | | | | | | | | | | | | | | | | | | | | | | | | | | | | | | | | | | | | | | | | | | | | | | | | | | | | | | |  | | | | | | | |  | | | | |  | | | | | |
| ( 5 ) Médico particular ( 6 ) Médico convênio ( 7 ) Em casa  ( 8 ) Outro, especificar:____________________________________ | | | | | | | | | | | | | | | | | | | | | | | | | | | | | | | | | | | | | | | | | | | | | | | | | | | | | | | |  | | | | | | | |  | | | | |  | | | | | |
| **53.6 Você foi orientado pelo serviço de saúde sobre algum tratamento NÃO medicamentoso,**  **que deve seguir para controlar a pressão?**(ASSINALAR AS ALTERNATIVAS RELATADAS ESPONTANEAMENTE PELO ENTREVISTADO).  ( 1 ) Sim ( 2 ) Não | | | | | | | | | | | | | | | | | | | | | | | | | | | | | | | | | | | | | | | | | | | | | | | | | | | | | | | |  | | | | | | | |  | | | | |  | | | | | |
|  |  |  |  |  |  |  |  |  |  |  |  |  |  |  |  |  |  |  |  |  |  |  |  |  |  |  |  |  |  |  |  |  |  |  |  |  |  |  |  |  |  |  |  |  |  |  |  |  |  |  |  |  |  |  |  |  | | | | | | | |  | | | | |  | | | | | |
|  |  | |  |  | |  | | |  | |  |  | | |  | | |  | | | | | | | | | |  | | | | | |  | | | | | | | |  | | | | | | | | | | | | | |  | | | | | | | |  | | | | |  | | | | | |
| **53.6.1 Sim, especificar:** ( 1 ) Diminuição da ingestão de sal ( 2 ) Perda de peso | | | | | | | | | | | | | | | | | | | | | | | | | | | | | | | | | | | | | | | | | | | | | | | | | | | | | | | |  | | | | | | | |  | | | | |  | | | | | |
| ( 3 ) Praticar exercícios físicos ( 4 ) Homeopatias  ( 5 ) Chás/ remédios caseiros tipo: _______________________________ | | | | | | | | | | | | | | | | | | | | | | | | | | | | | | | | | | | | | | | | | | | | | | | | | | | | | | | |  | | | | | | | |  | | | | |  | | | | | |
| ( 6 ) Meditação ( 8 ) Religiosidade/espiritualidade  ( 9 ) Outros, especificar: _______________________________ | | | | | | | | | | | | | | | | | | | | | | | | | | | | | | | | | | | | | | | | | | | | | | | | | | | | | | | |  | | | | | | | |  | | | | |  | | | | | |
|  |  | |  |  | |  | | |  | |  |  | | |  | | |  | | | | | | | | | |  | | | | | |  | | | | | | | |  | | | | | | | | | | | | | |  | | | | | | | |  | | | | |  | | | | | |
| **53.7 Você sabe informar com que frequência você vai ao serviço de saúde ou médico para tratar**  **a pressão alta?**  ( 1 ) Sim ( 2 ) Não | | | | | | | | | | | | | | | | | | | | | | | | | | | | | | | | | | | | | | | | | | | | | | | | | | | | | | | |  | | | | | | | |  | | | | |  | | | | | |
|  |  | |  |  | |  | | |  | |  |  | | |  | | |  | | | | | | | | | |  | | | | | |  | | | | | | | |  | | | | | | | | | | | | | |  | | | | | | | |  | | | | |  | | | | | |
| **53.7.1 Sim, qual a frequência?** ( 1 ) Mensal ( 2 ) Semestral ( 3 ) Anual ( 4 )Outros, especificar: _______________________________ | | | | | | | | | | | | | | | | | | | | | | | | | | | | | | | | | | | | | | | | | | | | | | | | | | | | | | | |  | | | | | | | |  | | | | |  | | | | | |
|  |  | |  |  | |  | | |  | |  |  | | |  | | |  | | | | | | | | | |  | | | | | |  | | | | | | | |  | | | | | | | | | | | | | |  | | | | | | | |  | | | | |  | | | | | |
| **53.8 Você sabe informar quando foi a última vez que você foi ao serviço de saúde ou médico para o**  **tratamento da pressão arterial?** ( 1 ) Sim ( 2 ) Não | | | | | | | | | | | | | | | | | | | | | | | | | | | | | | | | | | | | | | | | | | | | | | | | | | | | | | | |  | | | | | | | |  | | | | |  | | | | | |
| **53.8.1 Se Sim, especificar:**_________(meses) (ANOTAR SE A RESPOSTA DO ITEM 53.8 FOR SIM) | | | | | | | | | | | | | | | | | | | | | | | | | | | | | | | | | | | | | | | | | | | | | | | | | | | | | | | |  | | | | | | | |  | | | | |  | | | | | |
| **54. O Sr/ Sra. deixou de comparecer ás consultas marcadas no último ano?** ( 1 ) Sim ( 2 ) Não | | | | | | | | | | | | | | | | | | | | | | | | | | | | | | | | | | | | | | | | | | | | | | | | | | | | | | | |  | | | | | | | |  | | | | |  | | | | | |
| **54.1 Se Sim, especificar quantas vezes:**_______ (ANOTAR SE A RESPOSTA DO ITEM 54 FOR SIM) | | | | | | | | | | | | | | | | | | | | | | | | | | | | | | | | | | | | | | | | | | | | | | | | | | | | | | | |  | | | | | | | |  | | | | |  | | | | | |
| **54.2 Por quais motivos?** (ANOTAR SE A RESPOSTA DO ITEM 54 FOR SIM) | | | | | | | | | | | | | | | | | | | | | | | | | | | | | | | | | | | | | | | | | | | | | | | | | | | | | | | |  | | | | | | | |  | | | | |  | | | | | |
| ( 1 ) Esquecimento ( 2 ) Falta de dinheiro ( 3 ) Falta companhia | | | | | | | | | | | | | | | | | | | | | | | | | | | | | | | | | | | | | | | | | | | | | | | | | | | | | | | |  | | | | | | | |  | | | | |  | | | | | |
| ( 4 ) Filhos/parentes ( 5 ) Dificuldade de locomoção ( 6 ) Trabalho  ( 7 ) Outros: _______________________________ | | | | | | | | | | | | | | | | | | | | | | | | | | | | | | | | | | | | | | | | | | | | | | | | | | | | | | | |  | | | | | | | |  | | | | |  | | | | | |
| **55. O Sr/ Sra. deixou de tomar o(s) medicamento (s) para o tratamento da pressão nas últimas**  **DUAS semanas?**  ( 1 ) Sim ( 2 ) Não | | | | | | | | | | | | | | | | | | | | | | | | | | | | | | | | | | | | | | | | | | | | | | | | | | | | | | | |  | | | | | | | |  | | | | |  | | | | | |
|  |  | |  |  | |  | | |  | |  |  | | |  | | |  | | | | | | | | | |  | | | | | |  | | | | | | | |  | | | | | | | | | | | | | |  | | | | | | | |  | | | | |  | | | | | |
| **55.1 Se Sim:** (ASSINALAR AS ALTERNATIVAS RELATADAS ESPONTANEAMENTE PELO ENTREVISTADO). | | | | | | | | | | | | | | | | | | | | | | | | | | | | | | | | | | | | | | | | | | | | | | | | | | | | | | | |  | | | | | | | |  | | | | |  | | | | | |
| ( 1 ) Efeitos indesejáveis ( 2 ) Esquecimento ( 3 ) Achou que a pressão estava boa | | | | | | | | | | | | | | | | | | | | | | | | | | | | | | | | | | | | | | | | | | | | | | | | | | | | | | | |  | | | | | | | |  | | | | |  | | | | | |
| (4) Só toma o remédio quando se sente mal (5) Não estava melhorando a pressão | | | | | | | | | | | | | | | | | | | | | | | | | | | | | | | | | | | | | | | | | | | | | | | | | | | | | | | |  | | | | | | | |  | | | | |  | | | | | |
| ( 6 ) Não se importa com o fato de ter pressão alta ( 7 ) Achou que estava curado | | | | | | | | | | | | | | | | | | | | | | | | | | | | | | | | | | | | | | | | | | | | | | | | | | | | | | | |  | | | | | | | |  | | | | |  | | | | | |
| ( 8 ) Desconhecia a maneira de tomar o remédio ( 9 ) Preço do remédio | | | | | | | | | | | | | | | | | | | | | | | | | | | | | | | | | | | | | | | | | | | | | | | | | | | | | | | |  | | | | | | | |  | | | | |  | | | | | |
| (10) Desconhecia que tinha que tomar o remédio continuamente | | | | | | | | | | | | | | | | | | | | | | | | | | | | | | | | | | | | | | | | | | | | | | | | | | | | | | | |  | | | | | | | |  | | | | |  | | | | | |
| (11) Achou que ter pressão alta não é grave (12) Mediu a pressão e ela estava boa | | | | | | | | | | | | | | | | | | | | | | | | | | | | | | | | | | | | | | | | | | | | | | | | | | | | | | | |  | | | | | | | |  | | | | |  | | | | | |
| (13) Desconhecia que tinha pressão alta (14) Outro, especificar________________ | | | | | | | | | | | | | | | | | | | | | | | | | | | | | | | | | | | | | | | | | | | | | | | | | | | | | | | |  | | | | | | | |  | | | | |  | | | | | |
| ____________________________________________________________________ | | | | | | | | | | | | | | | | | | | | | | | | | | | | | | | | | | | | | | | | | | | | | | | | | | | | | | | |  | | | | | | | |  | | | | |  | | | | | |
| **56. Você tem alguma dificuldade para fazer o tratamento com medicamentos para pressão?**  ( 1 ) Sim ( 2 ) Não | | | | | | | | | | | | | | | | | | | | | | | | | | | | | | | | | | | | | | | | | | | | | | | | | | | | | | | |  | | | | | | | |  | | | | |  | | | | | |
|  |  | |  |  | |  | | |  | |  |  | | |  | | |  | | | | | | | | | |  | | | | | |  | | | | | | | |  | | | | | | | | | | | | | |  | | | | | | | |  | | | | |  | | | | | |
| **56.1 Se Sim:** (ASSINALAR AS ALTERNATIVAS RELATADAS ESPONTANEAMENTE PELO ENTREVISTADO). | | | | | | | | | | | | | | | | | | | | | | | | | | | | | | | | | | | | | | | | | | | | | | | | | | | | | | | |  | | | | | | | |  | | | | |  | | | | | |
| ( 1 ) Efeitos indesejáveis ( 2 ) Esquecimento ( 3 ) Achou que a pressão estava boa | | | | | | | | | | | | | | | | | | | | | | | | | | | | | | | | | | | | | | | | | | | | | | | | | | | | | | | |  | | | | | | | |  | | | | |  | | | | | |
| (4) Só toma o remédio quando se sente mal (5) Não estava melhorando a pressão | | | | | | | | | | | | | | | | | | | | | | | | | | | | | | | | | | | | | | | | | | | | | | | | | | | | | | | |  | | | | | | | |  | | | | |  | | | | | |
| ( 6 ) Não se importa com o fato de ter pressão alta ( 7 ) Achou que estava curado | | | | | | | | | | | | | | | | | | | | | | | | | | | | | | | | | | | | | | | | | | | | | | | | | | | | | | | |  | | | | | | | |  | | | | |  | | | | | |
| ( 8 ) Desconhecia a maneira de tomar o remédio ( 9 ) Preço do remédio | | | | | | | | | | | | | | | | | | | | | | | | | | | | | | | | | | | | | | | | | | | | | | | | | | | | | | | |  | | | | | | | |  | | | | |  | | | | | |
| (10) Desconhecia que tinha que tomar o remédio continuamente | | | | | | | | | | | | | | | | | | | | | | | | | | | | | | | | | | | | | | | | | | | | | | | | | | | | | | | |  | | | | | | | |  | | | | |  | | | | | |
| (11) Achou que ter pressão alta não é grave (12) Mediu a pressão e ela estava boa | | | | | | | | | | | | | | | | | | | | | | | | | | | | | | | | | | | | | | | | | | | | | | | | | | | | | | | |  | | | | | | | |  | | | | |  | | | | | |
| (13) Desconhecia que tinha pressão alta (14) Outro, especificar________________ | | | | | | | | | | | | | | | | | | | | | | | | | | | | | | | | | | | | | | | | | | | | | | | | | | | | | | | |  | | | | | | | |  | | | | |  | | | | | |
| ____________________________________________________________________ | | | | | | | | | | | | | | | | | | | | | | | | | | | | | | | | | | | | | | | | | | | | | | | | | | | | | | | |  | | | | | | | |  | | | | |  | | | | | |
|  |  | |  |  | |  | | |  | |  |  | | |  | | |  | | | | | | | | | |  | | | | | |  | | | | | | | |  | | | | | | | | | | | | | |  | | | | | | | |  | | | | |  | | | | | |
| **57. Qual a nota daria, de 1 a 5, para gravidade da pressão alta, considerando 1 uma doença sem**  **gravidade e 5 como de máxima gravidade?** | | | | | | | | | | | | | | | | | | | | | | | | | | | | | | | | | | | | | | | | | | | | | | | | | | | | | | | |  | | | | | | | |  | | | | |  | | | | | |
| ( 1 ) ( 2 ) ( 3 ) ( 4 ) ( 5 ) | | | | | | | | | | | | | | | | | | | | | | | | | | | | | | | | | | | | | | | | | | | | | | | | | | | | | | | |  | | | | | | | |  | | | | |  | | | | | |
|  |  | |  |  | |  | | |  | |  |  | | |  | | |  | | | | | | | | | |  | | | | | |  | | | | | | | |  | | | | | | | | | | | | | |  | | | | | | | |  | | | | |  | | | | | |
| **58. Você acha que ter pressão alta pode trazer complicações?** ( 1 ) Sim ( 2 ) Não ( 3 ) Não sabe | | | | | | | | | | | | | | | | | | | | | | | | | | | | | | | | | | | | | | | | | | | | | | | | | | | | | | | |  | | | | | | | |  | | | | |  | | | | | |
| **58.1 Sim, quais as complicações que a pressão alta pode trazer?**  (ASSINALAR AS ALTERNATIVAS RELATADAS ESPONTANEAMENTE PELO ENTREVISTADO). | | | | | | | | | | | | | | | | | | | | | | | | | | | | | | | | | | | | | | | | | | | | | | | | | | | | | | | |  | | | | | | | |  | | | | |  | | | | | |
| ( 1 ) Derrame/AVC ( 2 ) Infarto ( 3 ) Problemas no Rim | | | | | | | | | | | | | | | | | | | | | | | | | | | | | | | | | | | | | | | | | | | | | | | | | | | | | | | |  | | | | | | | |  | | | | |  | | | | | |
| ( 4 ) Outros, especificar:_____________________________________ | | | | | | | | | | | | | | | | | | | | | | | | | | | | | | | | | | | | | | | | | | | | | | | | | | | | | | | |  | | | | | | | |  | | | | |  | | | | | |
|  |  | |  |  | |  | | |  | |  |  | | |  | | |  | | | | | | | | | |  | | | | | |  | | | | | | | |  | | | | | | | | | | | | | |  | | | | | | | |  | | | | |  | | | | | |
| **59. Você sabe qual a partir de qual VALOR a pressão arterial é considerada ALTA?**  (ANOTAR COMO RELATADO PELO ENTREVISTADO). ( 1 ) Sim ( 2 ) Não | | | | | | | | | | | | | | | | | | | | | | | | | | | | | | | | | | | | | | | | | | | | | | | | | | | | | | | |  | | | | | | | |  | | | | |  | | | | | |
| **59.1 Se SIM, qual o valor?** Sistólica:_________mmHg X Diatólica:________mmHg | | | | | | | | | | | | | | | | | | | | | | | | | | | | | | | | | | | | | | | | | | | | | | | | | | | | | | | |  | | | | | | | |  | | | | |  | | | | | |
| **60. Na sua opinião a pressão alta pode ser curada?** ( 1 ) Sim ( 2 ) Não ( 3 ) Não sabe | | | | | | | | | | | | | | | | | | | | | | | | | | | | | | | | | | | | | | | | | | | | | | | | | | | | | | | |  | | | | | | | |  | | | | |  | | | | | |
|  |  | |  |  | |  | | |  | |  |  | | |  | | |  | | | | | | | | | |  | | | | | |  | | | | | | | |  | | | | | | | | | | | | | |  | | | | | | | |  | | | | |  | | | | | |
| **61. Por quanto tempo você acha que deve ser feito o tratamento para a pressão alta?** | | | | | | | | | | | | | | | | | | | | | | | | | | | | | | | | | | | | | | | | | | | | | | | | | | | | | | | |  | | | | | | | |  | | | | |  | | | | | |
| ( 1 ) Menos de 1 ano ( 2 ) De 1 a 10 anos ( 3 ) Para toda vida ( 4 ) Não sabe  ( 5 ) Outro: ________________________ | | | | | | | | | | | | | | | | | | | | | | | | | | | | | | | | | | | | | | | | | | | | | | | | | | | | | | | |  | | | | | | | |  | | | | |  | | | | | |

| **Questionário Internacional de Atividade Física- IPAQ Versão curta** | | | | | | | | | | | | | | | |
| --- | --- | --- | --- | --- | --- | --- | --- | --- | --- | --- | --- | --- | --- | --- | --- |
|  |  |  |  |  |  |  |  |  |  |  |  |  |  |  |  |
|  |  |  |  |  |  |  |  |  |  |  |  |  |  |  |  |
|  |  |  |  |  |  |  |  |  |  |  |  |  |  |  |  |
| **62. Em quantos dias da última semana você CAMINHOU por pelo menos 10 minutos contínuos em casa ou no trabalho, como forma de transporte para ir de um lugar para outro, por lazer, por prazer ou como forma de exercício?**  ( 1 ) Dias _______ por SEMANA ( 2 ) Nenhum | | | | | | | | | | | | | | | |
| **62.1 Nos dias em que você caminhou por pelo menos 10 minutos contínuos quanto tempo no total você gastou caminhando por dia?** ( 1 ) Horas: ______ Minutos: ______ | | | | | | | | | | | | | | | |
| **63. Em quantos dias da última semana, você realizou atividades MODERADAS por pelo menos 10 minutos contínuos, como por exemplo, pedalar leve na bicicleta, nadar, dançar, fazer ginástica aeróbica leve, jogar vôlei recreativo, carregar pesos leves, fazer serviços domésticos na casa, no quintal ou no jardim como varrer, aspirar, cuidar do jardim, ou qualquer atividade que fez aumentar moderadamente sua respiração ou batimentos do coração** (POR FAVOR NÃO INCLUA CAMINHADA) ( 1 ) Dias _______ por SEMANA ( 2 ) Nenhum | | | | | | | | | | | | | | | |
| **63.1 Nos dias em que você fez essas atividades moderadas por pelo menos 10 minutos contínuos, quanto tempo no total você gastou fazendo essas atividades por dia?** ( 1 ) Horas: ______ Minutos: _____ | | | | | | | | | | | | | | | |
| **64. Em quantos dias da última semana, você realizou atividades VIGOROSAS por pelo menos 10 minutos contínuos, como por exemplo correr, fazer ginástica aeróbica, jogar futebol, pedalar rápido na bicicleta, jogar basquete, fazer serviços domésticos pesados em casa, no quintal ou cavoucar no jardim, carregar pesos elevados ou qualquer atividade que fez aumentar MUITO sua respiração ou batimentos do coração.** ( 1 ) Dias _____ por SEMANA ( 2 ) Nenhum | | | | | | | | | | | | | | | |
|  |  |  |  |  |  |  |  |  |  |  |  |  |  |  |  |
| **64.1 Nos dias em que você fez essas atividades vigorosas por pelo menos 10 minutos contínuos quanto tempo no total você gastou fazendo essas atividades por dia?** ( 1 ) Horas: ______ Minutos: _____ | | | | | | | | | | | | | | | |
| **65. Estas últimas questões são sobre o tempo que você permanece sentado todo dia, no trabalho, na escola ou faculdade, em casa e durante seu tempo livre. Isto inclui o tempo sentado estudando, sentado enquanto descansa, fazendo lição de casa visitando um amigo, lendo, sentado ou deitado assistindo TV. Não inclua o tempo gasto sentando durante o transporte em ônibus ou carro.** ( 1 ) Horas: ______ Minutos: _____ | | | | | | | | | | | | | | | |
| **66. Quanto tempo por dia você fica sentado no final de semana?**  ( 1 ) Horas: ______ Minutos: _____ | | | | | | | | | | | | | | | |
|  |  |  |  |  |  |  |  |  |  |  |  |  |  |  |  |
|  |  |  |  |  |  |  |  |  |  |  |  |  |  |  |  |
| **Alcohol Use Disorders Identification – AUDIT** | | | | | | | | | | | | | | | |
|  |  |  |  |  |  |  |  |  |  |  |  |  |  |  |  |
|  |  |  |  |  |  |  |  |  |  |  |  |  |  |  |  |
|  |  |  |  |  |  |  |  |  |  |  |  |  |  |  |  |
| **67. Com qual frequência o Sr(a) utiliza bebidas com álcool ?** | | | | | | | | | | | | | | | |
| ( 0 ) Nunca ( 1 ) Uma vez por mês ou menos  ( 2 ) 2-4 vezes ao mês ( 3 ) 1-3 vezes por semana  ( 4 ) 4 ou mais vezes por semana | | | | | | |  |  |  |  |  |  |  |  |  |
|  |  |  |  |  |  |  |  |  |  |  |  |  |  |  |  |
| **68. Nas ocasiões em que bebe, quantas doses o Sr(a). costuma tomar ?** | | | | | | | | | | | | | | | |
| ( 0 ) 1 ou 2 doses ( 1 ) 3 ou 4 doses ( 2 ) 5 ou 6 doses  ( 3 ) 7 a 9 doses ( 4 ) 10 ou mais doses | | | | | | |  |  |  |  |  |  |  |  |  |
|  |  |  |  |  |  |  |  |  |  |  |  |  |  |  |  |
|  |  |  |  |  |  |  |  |  |  |  |  |  |  |  |  |
| **69. Com que frequência toma mais que 6 doses em uma única ocasião ?** | | | | | | | | | | | | | | | |
| ( 0 ) Nunca ( 1 ) Menos de uma vez ao mês  ( 2 ) Uma vez ao mês ( 3 ) Uma vez por semana  ( 4 ) Todos os dias ou quase todos | | | | | | |  |  |  |  |  |  |  |  |  |
|  |  |  |  |  |  |  |  |  |  |  |  |  |  |  |  |
|  |  |  |  |  |  |  |  |  |  |  |  |  |  |  |  |
| **70. Com que frequência no último ano o Sr(a) se sentiu incapaz de parar de beber depois que começou?** | | | | | | | | | | | | | | | |
| ( 0 ) Nunca ( 1 ) Menos de uma vez ao mês  ( 2 ) Uma vez ao mês ( 3 ) Uma vez por semana  ( 4 ) Todos os dias ou quase todos | | | | | | |  |  |  |  |  |  |  |  |  |
|  |  |  |  |  |  |  |  |  |  |  |  |  |  |  |  |
|  |  |  |  |  |  |  |  |  |  |  |  |  |  |  |  |
| **71. Com que frequência no último ano o Sr(a) não conseguiu cumprir com algum compromisso por causa da bebida ?** | | | | | | | | | | | | | | | |
| ( 0 ) Nunca ( 1 ) Menos de uma vez ao mês  ( 2 ) Uma vez ao mês ( 3 ) Uma vez por semana  ( 4 ) Todos os dias ou quase todos | | | | | | |  |  |  |  |  |  |  |  |  |
|  |  |  |  |  |  |  |  |  |  |  |  |  |  |  |  |
|  |  |  |  |  |  |  |  |  |  |  |  |  |  |  |  |
| **72. Com que frequência, durante o último ano, depois de ter bebido muito, o Sr(a) precisou beber pela manhã para se sentir melhor?** | | | | | | | | | | | | | | | |
| ( 0 ) Nunca ( 1 ) Menos de uma vez ao mês  ( 2 ) Uma vez ao mês ( 3 ) Uma vez por semana  ( 4 ) Todos os dias ou quase todos | | | | | | |  |  |  |  |  |  |  |  |  |
|  |  |  |  |  |  |  |  |  |  |  |  |  |  |  |  |
|  |  |  |  |  |  |  |  |  |  |  |  |  |  |  |  |
| **73. Com que frequência no último ano o Sr(a) sentiu culpa ou remorso após beber ?** | | | | | | | | | | | | | | | |
| ( 0 ) Nunca ( 1 ) Menos de uma vez ao mês  ( 2 ) Uma vez ao mês ( 3 ) Uma vez por semana  ( 4 ) Todos os dias ou quase todos | | | | | | |  |  |  |  |  |  |  |  |  |
|  |  |  |  |  |  |  |  |  |  |  |  |  |  |  |  |
| **74. Com que frequência no último ano o Sr(a) não conseguiu se lembrar o que aconteceu na noite anterior por causa da bebida ?** | | | | | | | | | | | | | | | |
| ( 0 ) Nunca ( 1 ) Menos de uma vez ao mês  ( 2 ) Uma vez ao mês ( 3 ) Uma vez por semana  ( 4 ) Todos os dias ou quase todos | | | | | | |  |  |  |  |  |  |  |  |  |
|  |  |  |  |  |  |  |  |  |  |  |  |  |  |  |  |
|  |  |  |  |  |  |  |  |  |  |  |  |  |  |  |  |
| **75. O Sr(a) já se machucou ou machucou alguém como resultado do seu uso de álcool ?** | | | | | | | | | | | | | | | |
| ( 0 ) Não ( 2 ) Sim, mas não no último ano  ( 4 ) Sim, durante o último ano | | | | | | |  |  |  |  |  |  |  |  |  |
|  |  |  |  |  |  |  |  |  |  |  |  |  |  |  |  |
| **76. Algum parente ou amigo ou médico ou outro profissional de saúde se preocupou com seu hábito ou sugeriu que parasse de beber ?** | | | | | | | | | | | | | | | |
| ( 0 ) Não ( 2 ) Sim, mas não no último ano  ( 4 ) Sim, durante o último ano | | | | | | |  |  |  |  |  |  |  |  |  |
|  |  |  |  |  |  |  |  |  |  |  |  |  |  |  |  |
|  |  |  |  |  |  |  |  |  |  |  |  |  |  |  |  |
| *Preencha as questões 2 e 3 transformando as quantidades em “doses”, baseado no quadro abaixo.* | | | | | | | | | | | | | | | |
| CERVEJA: 1 copo (de chopp – 350 ml), 1 lata – 1 “dose” ou garrafa – 2 “doses” VINHO: 1 copo comum grande (250 ml) – 2 doses ou garrafa – 8 doses CACHAÇA, VODCA, UÍSQUE ou CONHAQUE: 1 “martelinho” (60ml) – 2 doses; 1 “martelo”(100ml) – 3 doses ou 1 garrafa – mais de 20 doses UÍSQUE, RUM, LICOR, etc: 1 dose de dosador (45-50 ml) – 1 dose | | | | | | | | | | | | | | | |
|  |  |  |  |  |  |  |  |  |  |  |  |  |  |  |  |
|  |  |  |  |  |  |  |  |  |  |  |  |  |  |  |  |
|  |  |  |  |  |  |  |  |  |  |  |  |  |  |  |  |
|  |  |  |  |  |  |  |  |  |  |  |  |  |  |  |  |
|  |  |  |  |  |  |  |  |  |  |  |  |  |  |  |  |
| **77. Com que frequência o Sr (a) beber o caxiri?**  ( 0 ) Nunca ( 1 ) Menos de uma vez ao mês  ( 2 ) Uma vez ao mês ( 3 ) Uma vez por semana  ( 4 ) Todos os dias ou quase todos  **78. Qual a quantidade que o Sr (a) bebe o caxiri?**  ( 0 ) 1 copo ( 1 ) cuia  ( 2 ) 1 lata ( 3) garrafa pet 1 litro | | | | | | | | | | | | | | | |
| **Self Report Questionnaire (SQR 20)** | | | | | | | | | | | | | | | |
|  |  |  |  |  |  |  |  |  |  |  |  |  |  |  |  |
|  |  |  |  |  |  |  |  |  |  |  |  |  |  |  |  |
|  |  |  |  |  |  |  |  |  |  |  |  |  |  |  |  |
| **O(A) Sr.(a) teve algum destes problemas nos últimos 30 dias?** | | | | | **Não** | | | **Sim** | | |  |  |  |  |  |
| 79. Tem dores de cabeça frequentes?. | | | | | 0 | | | 1 | | |  |  |  |  |  |
| 80. Tem falta de apetite?. | | | | | 0 | | | 1 | | |  |  |  |  |  |
| 81. Dorme mal? | | | | | 0 | | | 1 | | |  |  |  |  |  |
| 82. Assusta-se com facilidade? | | | | | 0 | | | 1 | | |  |  |  |  |  |
| 83. Tem tremores de mão? | | | | | 0 | | | 1 | | |  |  |  |  |  |
| 84. Sente-se nervoso(a), tenso(a) ou preocupado(a) | | | | | 0 | | | 1 | | |  |  |  |  |  |
| 85. Tem má digestão? | | | | | 0 | | | 1 | | |  |  |  |  |  |
| 86. Tem dificuldade de pensar com clareza? | | | | | 0 | | | 1 | | |  |  |  |  |  |
| 87. Tem se sentido triste ultimamente? | | | | | 0 | | | 1 | | |  |  |  |  |  |
| 88. Tem chorado mais do que de costume? | | | | | 0 | | | 1 | | |  |  |  |  |  |
| 89. Encontra dificuldades para realizar com satisfação suas atividades diárias? | | | | | 0 | | | 1 | | |  |  |  |  |  |
| 90. Tem dificuldades para tomar decisões? | | | | | 0 | | | 1 | | |  |  |  |  |  |
| 91. Tem dificuldades no serviço (seu trabalho é penoso, causa sofrimento)? | | | | | 0 | | | 1 | | |  |  |  |  |  |
| 92. É incapaz de desempenhar um papel útil em sua vida? | | | | | 0 | | | 1 | | |  |  |  |  |  |
| 93. Tem perdido o interesse pelas coisas? | | | | | 0 | | | 1 | | |  |  |  |  |  |
| 94.Você se sente uma pessoa inútil, sem préstimo? | | | | | 0 | | | 1 | | |  |  |  |  |  |
| 95.Tem tido ideias de acabar com a vida | | | | | 0 | | | 1 | | |  |  |  |  |  |
| 96. Sente-se cansado(a) o tempo todo? | | | | | 0 | | | 1 | | |  |  |  |  |  |
| 97. Tem sensações desagradáveis no estômago? | | | | | 0 | | | 1 | | |  |  |  |  |  |
| 98. Você se cansa com facilidade? | | | | | 0 | | | 1 | | |  |  |  |  |  |

Anotações Gerais

_____________________________________________________________________________________

_____________________________________________________________________________________

_____________________________________________________________________________________

_____________________________________________________________________________________

_____________________________________________________________________________________

_____________________________________________________________________________________

_____________________________________________________________________________________

_____________________________________________________________________________________

_____________________________________________________________________________________

_____________________________________________________________________________________

_____________________________________________________________________________________

_____________________________________________________________________________________

_____________________________________________________________________________________

_____________________________________________________________________________________

_____________________________________________________________________________________

_____________________________________________________________________________________

_____________________________________________________________________________________

_____________________________________________________________________________________

_____________________________________________________________________________________

_____________________________________________________________________________________

_____________________________________________________________________________________

_____________________________________________________________________________________

_____________________________________________________________________________________

_____________________________________________________________________________________

_____________________________________________________________________________________

_____________________________________________________________________________________

_____________________________________________________________________________________

_____________________________________________________________________________________

_____________________________________________________________________________________

_____________________________________________________________________________________

_____________________________________________________________________________________

_____________________________________________________________________________________

_____________________________________________________________________________________

_____________________________________________________________________________________

_____________________________________________________________________________________

_____________________________________________________________________________________

_____________________________________________________________________________________

_____________________________________________________________________________________

_____________________________________________________________________________________

_____________________________________________________________________________________

_____________________________________________________________________________________

_____________________________________________________________________________________

_____________________________________________________________________________________

_____________________________________________________________________________________

_____________________________________________________________________________________

_____________________________________________________________________________________

_____________________________________________________________________________________

_____________________________________________________________________________________

_____________________________________________________________________________________

_____________________________________________________________________________________

_____________________________________________________________________________________

_____________________________________________________________________________________

_____________________________________________________________________________________

_____________________________________________________________________________________

_____________________________________________________________________________________

_____________________________________________________________________________________

_____________________________________________________________________________________

_____________________________________________________________________________________

_____________________________________________________________________________________

_____________________________________________________________________________________

_____________________________________________________________________________________

_____________________________________________________________________________________

_____________________________________________________________________________________

_____________________________________________________________________________________

_____________________________________________________________________________________

_____________________________________________________________________________________

_____________________________________________________________________________________

_____________________________________________________________________________________

_____________________________________________________________________________________

_____________________________________________________________________________________

_____________________________________________________________________________________

_____________________________________________________________________________________

_____________________________________________________________________________________

_____________________________________________________________________________________

_____________________________________________________________________________________

_____________________________________________________________________________________
